# Vibrational Spectroscopy and Computational Studies of Polyhalogenated Ferrocenes

**DOI:** 10.3390/molecules31193461

**Published:** 2026-09-28

**Authors:** Stewart F. Parker, Keith H. Hughes, Ian R. Butler

**Affiliations:** 1 ISIS Neutron and Muon Facility, STFC Rutherford Appleton Laboratory, Chilton OX11 0QX, Oxfordshire, UK; 2 Department of Chemistry, Bangor University, Bangor LL57 2TW, Gwynedd, UK; khhuws@gmail.com (K.H.H.); i.r.butler@hotmail.co.uk (I.R.B.)

**Keywords:** ferrocene, polyhalogenated ferrocene, inelastic neutron scattering spectroscopy, infrared spectroscopy, Raman spectroscopy, density functional theory

## Abstract

In this work we have provided the first comprehensive investigation of the vibrational spectra of polyhalogenated haloferrocenes; viz 1,1′-dibromoferrocene, 1,1′,2,2′-tetrabromoferrocene, 1,1′,2,2′,3,3′-hexabromoferrocene and 1,1′,2,2′-tetraiodoferrocene. These are characterised by infrared, Raman and inelastic neutron scattering (INS) spectroscopies supported by periodic density functional theory calculations of the primitive cell. The spectra differ from those of ferrocene in the region below 500 cm^−1^ because the C–Br and C–I modes occur here. The modes in this region include the frustrated rotational and translational modes of the iron atom and the Cp rings. Some of these are present as whole-molecule librations and translations below 100 cm^−1^ but some become intramolecular modes of the entire molecule—ring tilts and bends—and these are mass-dependent.

## 1. Introduction

The discovery of ferrocene (biscyclopentadienyl iron (II), Fe(η^5^-C_5_H_5_)_2_) [1,2] in the early 1950s and the subsequent recognition of its sandwich structure [3,4] can be said to be the start of modern organometallic chemistry [5,6].

Since those early days, the chemistry of ferrocene has massively expanded to include applications in organic synthesis, homogeneous catalysis and materials science [7]. This arises from the facile substitution of the cyclopentadienyl rings to form an essentially unlimited number of possible derivatives [8]. Present uses for ferrocene derivatives include medicine (ferroquine and ferrocifen for the treatment of malaria and cancer, respectively; the former is in phase II clinical trials [9]), as ligands involved in the synthesis of Perspex™ (the Alpha Process [10]), enantioselective reactions (via chiral ferrocene-based phosphines [11]) and potential usage in sensors and as a rocket fuel additive [12]. Ferrocene (and its derivatives) are a significant commercial enterprise: in 2024 the market size was valued at $71.39 M [13].

The structure and vibrational spectroscopy of ferrocene have been the subject of extensive study since its first synthesis. In particular, whether the cyclopentadienyl rings are eclipsed (the carbon atoms lie above each other, *D*_5h_ conformer) or staggered (the carbon atoms are above the mid-point of the C–C bonds of the second ring, *D*_5d_ conformer), the current view is that the conformer that is found (this can be *D*_5h_, *D*_5d_ or *C*_1_) depends on the temperature and pressure of the measurement [14]. The history is comprehensively reviewed in [15].

A subgroup of ferrocene derivatives that have proven especially useful are the halogenated ferrocenes, as these provide entries for lithiated derivatives that are precursors for many more complex systems [16,17]. There are a large number of possible haloferrocenes: as noted elsewhere [18], “if only homohaloferrocenes are considered, there are 35 compounds for each of the four halogens possible (1 mono-, nona- and decahaloferrocene each, 3 di-and octahaloferrocenes each, 4 tri- and heptahaloferrocenes each and 6 tetra-, penta- and hexahaloferrocenes each). Most of the homohaloferrocenes are unpublished: as of 2018 only 7 fluoro-, 12 chloro-, 10 bromo- and 8 iodoferrocenes have been prepared.” Of the 37 known materials, there are structures for 21 of them (3 F, 6 Cl, 7 Br, 5 I) in the Cambridge Structural Database [19].

The synthetic route to obtain these compounds generally involves metalation of the ferrocene followed by quenching with a halogen source. In theory, the synthetic methods currently available can produce every isomer, but in practice, inseparable mixtures (at scale) are frequently obtained. The reason that there are fewer fluoro- compounds reported is due to the lack of a reliable highly soluble quenching reagent. In contrast, chloroferrocenes are the most easily obtained and do not present a synthetic challenge as the carbon chlorine bond is more stable under metalation reaction conditions, so product isomerisation is not common. Conceptually, all 35 bromoferrocenes are accessible using the current lithiation route. In general, where pure single isomers are required, an iterative synthetic approach is taken. As an example, 1,1′,2,2′,3,3′-hexabromoferrocene is obtained from an approximately equal mixture of the corresponding 1,1′,2,2′-tetrabromo-3,3′-dilithio- and 1,1′,2,2′-tetrabromo-3,5′-dilithio ferrocenes which are prepared from 1,1′,2,2′-tetrabromoferroce in non-polar solvents (e.g., hexanes). If the intermediate lithiated bromoferrocenes are added to more polar solvents, such as THF, isomerisation occurs and upon quenching these isomers produce more complex mixtures (via the halogen dance) of the other hexabromoferrocene isomers. It is noted that even in non-polar solvents, partial bromine occupancy is observed in the product hexabromoferrocene crystal structure because the intermediate lithiobromoferrocenes are diastereotopic (see Figure 1).

The remaining hexabromoferrocenes, (1,1′,2,3,4,5-, (5,1), 1,1′,2,2′,3,4-, (4,2), 1,1′,2,3,3′4-, (4,2), 1,1′,2,2′,3,4′-, (3,3), and 1,1′,2,2′4,4′- (3,3)- could individually be prepared from suitable bromoferrocene precursors by mono- or dilithiation. [Here, (x,y) refers to the number of bromine atoms on each ring]. A similar logic may be applied in the syntheses of the bromoferrocenes with 3–8 bromine atoms using the alpha lithiation methodology. Further structural details of a series of highly substituted bromoferrocenes have been reported, ref. [20] including 1,1′,2,2′,3,3′-hexabromoferrocene, prepared under modified conditions, and decabromoferrocene [21]. It is clear that when more polar solvents are used in these reactions that the actual synthesis may not be a challenge, but the clean separation of isomeric products clearly is. Iodoferrocenes, in turn, pose the most difficult synthetic challenge using metalation/exchange routes because of their metalated intermediates’ tendency towards Wurtz coupling and their ease of metal–halogen exchange which can result in subsequent intermolecular exchange.

The vibrational spectroscopy of ferrocene has been comprehensively studied both experimentally [22,23,24,25,26,27] and computationally [24,28,29,30]. In contrast, the spectroscopy of substituted ferrocenes is sparse. Several ferrocenes with (poly)methylated cyclopentadienyl rings [31,32,33,34] have been studied, as have the 1,1′-dihaloferrocenes (halo = Cl, Br, I) [35]. We are unaware of any other studies where the focus is the detailed assignment of modes rather than routine characterisation.

Recent work [16,17,20] has provided access to structurally characterised polyhalogenated ferrocenes and these provide an opportunity to investigate how changes in the cyclopentadienyl rings manifest in the spectra. Specifically, 1,1′-dibromoferrocene [36], 1,1′,2,2′-tetrabromoferrocene [16], 1,1′,2,2′,3,3′-hexabromoferrocene [20] and 1,1′,2,2′-tetraiodoferrocene [37] are characterised by infrared, Raman and inelastic neutron scattering (INS) spectroscopies supported by periodic density functional theory calculations of the primitive cell.

## 2. Results and Discussion

We will discuss each compound individually and then examine trends in the spectra.

### 2.1. 1,1′-Dibromoferrocene

1,1′-Dibromoferrocene crystallises in the monoclinic space group *P*2_1_ (no. 4) with two molecules in the primitive cell, both on *C*_1_ sites [36]. As seen in Figure 2, the bromine atoms are eclipsed, which results in approximate *C*_2v_ symmetry.

The infrared, Raman and INS spectra of 1,1′-dibromoferrocene are shown in Figure 3. INS is a form of vibrational spectroscopy [38] where scattering occurs between the neutron and the atomic nucleus, mediated by the nuclear strong force. This has the consequence that (except in very special circumstances [39]) there are no selection rules, so all modes are formally allowed. The intensity of a mode depends on the amplitude of motion of the atoms involved in the mode and their neutron scattering cross-section [38]. For the present case, the relevant cross-sections are as follows: ^1^H = 82.03, ^2^H = 7.64, C = 5.55, Fe = 11.62, Br = 5.90, I = 3.81 barn (1 barn = 10^−28^ m^2^). As ^1^H has both the smallest mass (and hence largest amplitude of motion) and the largest cross-section, it follows that the INS spectra are dominated by modes that involve hydrogen motion. Note that it is only movement of hydrogen that is required; so not only modes that directly involve hydrogen, such as C–H stretch and bend modes, but also the low-energy modes that result in ring deformation or whole-body displacements of the rings result in strong INS modes. It is apparent that all three types of spectroscopy are needed for a complete description; modes that are strong in one form of spectroscopy may be weak or absent in the others.

While the assignment of some of the modes is straightforward, e.g., C–H stretching, C–C stretching, and C–H bending, the assignment of the Fe–ring modes is not. Computational spectroscopy offers a way forward here. Comparison of observed and calculated INS spectra is a stringent test of this calculation, as shown in Figure 4. There is very good agreement between the two spectra, enabling a complete assignment to be made.

### 2.2. 1,1′,2,2′-Tetrabromoferrocene

1,1′,2,2′-Tetrabromoferrocene crystallises in the monoclinic space group *P*2/*n* (no. 13) with two molecules in the primitive cell, both on *C*_2_ sites [16]. As seen in Figure 5, the bromine atoms are eclipsed, which results in approximate *C*_2v_ symmetry.

The infrared, Raman and INS spectra of 1,1′,2,2′-tetrabromoferrocene are shown in Figure 6 and the comparison between observed and calculated INS spectra is shown in Figure 7.

### 2.3. 1,1′,2,2′,3,3′-Hexabromoferrocene

1,1′,2,2′,3,3′-Hexabromoferrocene crystallises in the triclinic space group $P\bar{1}$ (no. 2) with two molecules in the primitive cell, both on *C*_1_ sites [20]. As seen in Figure 8, the bromine atoms are eclipsed, which results in approximate *C*_2v_ symmetry. This isomer is the majority species (96.4%), there is also an isomer where one ring is rotated by 72° relative to the second ring, so that one of the Br atoms in each ring is above a H atom in the other ring to give *C*_2_ symmetry. As this is only present to the extent of 3.6%, we neglect it and only consider the majority species.

The infrared, Raman and INS spectra of 1,1′,2,2′,3,3′-hexabromoferrocene are shown in Figure 9 and the comparison between observed and calculated INS spectra is shown in Figure 10.

### 2.4. 1,1′,2,2′-Tetraiodoferrocene

1,1′,2,2′-Tetraiodoferrocene crystallises in the monoclinic space group *P*2_1_/*n* (no. 14) with eight molecules in the primitive cell, all on *C*_1_ sites [37]. As shown in Figure 11, the atoms are eclipsed; however, the iodine atoms are above hydrogen atoms, which results in approximate *C*_2_ symmetry. This is a distinct difference to the Br-containing ferrocenes where the bromine atoms are always above each other.

The infrared, Raman and INS spectra of 1,1′,2,2′-tetraiodoferrocene are shown in Figure 12. Unexpectedly, the infrared spectrum, as shown in Figure 12a, contains aliphatic C–H modes (bands at 2800–3000 cm^−1^). This may be a residual trace of the hexane solvent. There is no evidence of this in either of the other spectra, indicating that the amount is negligible.

The comparison between observed and calculated INS spectra is shown in Figure 13. Owing to the large number of molecules in the primitive cell, in this one case, the calculation is for a single, isolated molecule (in a 15 × 15 × 15 Å box), rather than the complete unit cell. While this is unfortunate, the spectra show no evidence for factor group splitting, indicating that the intermolecular interactions are small. This is consistent with the closest contact being an H···H interaction at 2.6 Å. In fact, all interactions at less than 3.0 Å are of this type. The presence of eight molecules in the unit cell is most apparent in the low-energy (below 100 cm^−1^) region where the external modes are observed. The only internal mode that occurs in this region is the ring torsion and as the focus of this work is the internal modes, considering an isolated molecule (but one based on the crystal structure) is a reasonable approximation. The good agreement of the observed and calculated INS spectra at >200 cm^−1^ supports this conclusion.

### 2.5. Comparison of Spectra

Figure 14, Figure 15 and Figure 16 compare the INS, infrared and Raman spectra of the haloferrocenes with those of the parent molecule, ferrocene. In the INS spectra, as shown in Figure 14, above 550 cm^−1^, the spectra are similar to each other. The INS spectra are dominated by modes involving the motion of hydrogen. The animations show that the modes in the 800–900 cm^−1^ region are C–H out-of-plane bending modes and those in the 1000–1100 cm^−1^ region are the corresponding C–H in-plane bending modes. The bond lengths in ferrocene, as determined by neutron diffraction at 173 K, are 1.014–1.065 Å [40]. These are very close to those calculated for the haloferrocenes, which are all ~1.083 Å, so the transition energies for C–H modes are expected to be similar in all cases, as observed. (We cite calculated rather than experimental C–H distances for the haloferrocenes because these were determined by X-ray diffraction and this consistently underestimates the C–H distance [41]).

The mode at ~600 cm^−1^ is a cyclopentadienyl ring deformation. This remains at almost the same energy in all the systems because the large mass of the halo atoms means that they are largely immobile in the mode, so do not alter the carbon displacements that drive the mode.

In contrast, the region below 550 cm^−1^ shows little commonality between the spectra. Partly this is because the C–halo stretch and bend modes are in this region and because modes that involve whole-body motion of the rings vary with halo-substitution, and hence changing mass, of the cyclopentadienyl rings.

In contrast to the INS spectra, the infrared and Raman spectra, as shown in Figure 15 and Figure 16, of the haloferrocenes are distinctly different from those of ferrocene itself. In both cases the spectra are much more complex than ferrocene’s. This arises because the reduced symmetry of the haloferrocenes means that most modes are allowed in both the infrared and Raman spectra. While ferrocene below 164 K [42] is formally *C*_1_ symmetry, it is reasonably close to that of the idealised *D*_5h_ system so that the selection rules are largely followed, resulting in sparse spectra.

In the infrared spectra, the strong modes are also those seen in the INS, (ring deformation at ~500 cm^−1^ and 800–900 cm^−1^ for C–H out-of-plane bending modes) albeit with better resolution, so they appear as multiplets. The difference is the strong modes at 1300–1400 cm^−1^. These are assigned as C–C stretch modes; they appear in the infrared because the calculations show that the carbon atoms carry a substantial Mulliken charge (−0.290 to –0.406 electron) so displacement of these results in a substantial induced dipole and hence strong infrared activity. However, the amplitude of motion is relatively small, so the corresponding hydrogen motion is small, resulting in weak INS modes, as shown in Figure 14.

The Raman spectra are dominated by intense modes in the region below 500 cm^−1^; as stated earlier, this is where the C-halo and whole-body modes occur, as discussed in the next section.

The exception to this pattern is 1,1′-dibromoferrocene, whose spectrum approximates to that of ferrocene itself. Presumably, this is because replacement of only one hydrogen in each cyclopentadienyl ring does not greatly perturb the system, except for the reduction in symmetry.

### 2.6. Modes Involving Fe and the Halogens

The comparisons of the experimental and calculated INS spectra (Figure 4, Figure 7, Figure 10 and Figure 13) show excellent agreement, demonstrating that the calculations can provide robust assignments. The modes that are the most interesting are those that involve the Fe atom or the halogens and the calculations provide a means to investigate these. The AbINS programme [43] that is used to generate the calculated INS spectra from the computational output includes the contribution of each type of atom (H, C, Fe, halogen) to the INS spectrum. The individual atom-type spectra thus highlight the modes that involve motion of only that type of atom. Figure 16 shows the Fe-only and halogen-only spectra for ferrocene and the haloferrocenes. Table 1 lists the modes involving Fe and Table 2 those involving the halogens.

Iron is very unusual in that it is possible to carry out Nuclear Resonance Vibrational Spectroscopy (NRVS) [44] with it. This provides an atom-selective vibrational density of states when a Mössbauer active atomic nucleus, such as the ^57^Fe isotope, is present. The significance of this is that the spectrum only includes modes that involve motion of the Fe atom. Ferrocene has been measured by NRVS [25] and the spectrum is reproduced in Figure 17a. Three bands are apparent at 170, 470 and 490 cm^−1^. These were assigned as the asymmetric stretching of the Cp–Fe–Cp bonds and the bending of the Cp–Fe–Cp bond angle (Cp = cyclopentadienyl) respectively. In the ideal *D*_5h_, isolated molecule the latter is doubly degenerate, in the solid state the *C*_1_ symmetry [41] removes the degeneracy and this was given as why two modes are observed [25]. This assignment is incorrect because it treats the molecule as a simple triatomic. The near-*D*_5h_ symmetry means that there are five metal–ring vibrations: symmetric and asymmetric (with respect to the horizontal mirror plane in the isolated molecule), Fe–ring stretch (ν_4_, *A*_1_′ and ν_11_, *A*_2_″), Fe–ring tilt (ν_16_, *E*_1_″ and ν_21_, *E*_1_′) and the ring–Fe–ring bend (ν_22_, *E*_1_′) (the mode numbering used is that of [22,23] and the form of the modes is shown in [23]). Of these, three involve motion of the iron atom: ν_22_ (179 cm^−1^), ν_11_ (478 cm^−1^) and ν_21_ (492 cm^−1^) [22,23]. We can see that the calculated Fe-only spectrum of ferrocene, as shown in Figure 16a (blue trace), is in excellent agreement with the NRVS spectrum shown in Figure 16a (purple trace), providing confidence in the method.

It is apparent that the Fe-only spectra of the haloferrocenes are more complex than that of ferrocene itself. Partly this is because of the reduction in symmetry in the idealised molecules (there are no degenerate modes in molecules with *C*_2_ or *C*_2v_ symmetry). More importantly, to preserve the centre-of-mass of the unit cell (a requirement for a normal mode), movement of the heavy atoms, Br or I, results in movement of the Cp rings and the iron atom. A case in point is the weak feature at ~580 cm^−1^, which is present in all haloferrocene spectra. This is an out-of-plane ring deformation about an axis (in each ring) that includes the C–halo bond. In these compounds, the halogen “anchors” the ring so to compensate for this, the iron atom must move. This cannot happen in ferrocene itself, so there is no feature at ~580 cm^−1^ in Figure 17a.

Inspection of the spectra shows that the asymmetric stretch (ν_11_) and the ring tilt (ν_21_) remain fairly constant at ~500 cm^−1^, even though the mass of the rings increases from 65 each in ferrocene to 307 each in 1,1′,2,2′-tetraiodoferrocene. The mode visualisations show that this is because the halogens are largely immobile (as confirmed by the red traces that show halogen motion in Figure 17b–e, which are flat in this region); as such, they do not contribute to the modes, so the effective mass of each ring is ~60 in all cases, so the transition energy is unchanged.

In contrast, the doubly degenerate ring bend (ν_22_) splits into two components, one of which is approximately at the same energy as in ferrocene itself and the second, which is at somewhat higher energy. This is because in the lower energy mode, the ring motion does not involve the halogen whilst it does for the higher energy mode.

There is surprisingly little literature on the vibrational modes of carbon–halogen bonds. The standard texts, e.g., [45], largely only consider monohalaoalkanes and their conformational isomerism. The assignment of monosubstitutedbenzenes has been extensively debated for many years; Gardner and Wright [46] provided a comprehensive discussion of the various assignment schemes and produced the first consistent set of assignments for this group of compounds. Of relevance here, they produced complete assignments for the monohalobenzenes and these are given in Table 2 for bromo- and iodobenzene.

The same method used to isolate the Fe-related modes can be used for the halogens. Figure 17b–e and Table 2 show the results. The values for the haloferrocenes are similar to those found for bromo- and iodobenzene. This is reasonable as both benzene and Cp have six electron aromatic systems. It is noticeable that most of the halogen modes also involve motion of the iron.

The modes are best seen in the Raman and INS spectra. At first sight, strong Br-related modes in the INS data are surprising; however, as noted for the Fe-modes, movement of Br or I results in large motions of the Cp ring and consequently strong INS modes.

The most controversial mode of metallocenes is the ring torsion, where each ring rotates in the opposite direction to each other. In ruthenocene, the four-factor group components (there are four molecules in the primitive cell) are calculated to be at 42, 58, 110 and 114 cm^−1^ [47]. In ferrocene, the torsion has been assigned as 6 cm^−1^ [24] and 44 cm^−1^ [28] based on computational studies of the isolated molecule. The INS spectrum of ferrocene shows a sharp peak at 20 cm^−1^. In the Debye approximation, the low-energy vibrational density of states of a three-dimensional elastic solid is proportional to υ^2^, reflecting the linear dispersion of long-wavelength acoustic phonons [48]; thus, a mode would not be expected at 20 cm^−1^, so we assign this as the torsion in the solid state. Inspection of the mode visualisations for the haloferrocenes identified the torsion at: 1,1′-dibromoferrocene 55/55, 1,1′,2,2′-tetrabromoferrocene 57/64 cm^−1^, 1,1′,2,2′,3,3′-hexabromoferrocene 41/58 cm^−1^ and 1,1′,2,2′-tetraiodoferrocene 7 cm^−1^. (The last of these is unreliable because it does not include the intermolecular interactions that raise the transition energy). Inspection of the experimental INS spectra of the bromoferrocenes found peaks at these values; however, caution is needed because these are in the external density of states and exhibit vibrational dispersion (variations in transition energy with wavevector) and the calculation was conducted for the Brillouin zone Γ-point only, as the full dispersion calculation would be prohibitively expensive. (Unlike infrared and Raman spectroscopy, where the modes are only observed at the Brillouin zone Γ-point; i.e., at (almost) zero wavevector, INS can observe modes at all wavevectors. This is a consequence of the neutron having significant mass. Thus, modes that exhibit significant dispersion result in broad, and possibly structured, features in the INS spectrum, hence the need for caution in the low-energy region. The sharpness of the features in the internal mode region demonstrates that any dispersion is less than the line width.)

**Table 2:** Carbon–halogen modes.

Molecule	Stretch/cm^−1^	In-Plane Bend/cm^−1^	Out-of-Plane Bend/cm^−1^
1,1′-Dibromoferrocene	308, 323 ^1^	234, 245	84, 113
	(308–320)	(245)	(91–125)
1,1′,2,2′-Tetrabromoferrocene	332, 347	277	107, 136
	(328–348)	(264)	(101–144)
1,1′,2,2′,3,3′-Hexabromoferrocene	349, 359	236	104, 120
	(322–349)	(225)	(100–120)
1,1′,2,2′-Tetraiodoferrocene	285	208, 227	Not observed
	(280–307)	(207–218)	(56–88)
Bromobenzene [46]	315	254	181
Iodobenzene [46]	266 ^2^	220	181

^1^ The observed transition energies are given for the experimental values and the values in brackets are the highest and lowest value of the factor group components for the calculated spectra. ^2^ Only a single molecule was calculated for this molecule, so there are no factor group components.

### 2.7. Why Are the Bromine Atoms Eclipsed?

Of particular interest is the structure of 1,1′,1,2′-tetrabromoferrocene and its ruthenium analogue, 1,1′2,2′-tetrabromoruthenocene [16]. In both cases the structures exhibit an almost eclipsed structure; however, after preparing a significant number of compounds of this type where the halogen was varied [16,17,18,49], we found that this eclipsed structure is the norm. As noted earlier, the experimentally determined structure of 1,1′-dibromoferrocene from X-ray diffraction [36] is 0° eclipsed (syn-periplanar). However, the gas phase structure of 1,1′-dibromoferrocene, which is based on theoretical calculations and compared to experimental infrared studies of the isolated compound in an argon matrix, indicates that 72° (synclinical) and 144° (anticlinical) eclipsed structures are most stable [50]. Microwave spectroscopy studies on 1,1′-dimethylferrocene observed two eclipsed structures at eclipsed 0 and 72° [51]. However, as for the dibromo case, these studies also relied on theoretical calculations to verify the stable structures of the isolated molecule. To account for the eclipsed structure of 1,1′2,2′-tetrabromoferrocene, we carried out detailed calculations on the molecule itself.

Initial calculations were made using the X-ray structure coordinates of 1,1′2,2′-tetrabromoferrocene [16] with optimisation of the hydrogen atoms only. The Br substitution in adjacent positions on the Cp rings is expected to display some Br-Br dispersive interactions. The ‘cis’ geometry of the structure is also expected to display some dispersive interactions between the Br atoms on each ring. The topological visualisation of these weak non-covalent Br-Br interactions is performed using the so called ‘non-covalent interaction’ (NCI) approach, as implemented in the NCIPLOT programme [52], which is based on the reduced density gradient:

(1) $$s\left(r\right)=\frac{\left|\nabla \rho \left(r\right)\right|}{2(3\pi^{2}{)}^{\frac{1}{3}}\rho (r{)}^{4/3}}$$

where the charge density, $\rho \left(r\right)$, and its gradient, $\nabla \rho \left(r\right)$, is obtained from the DFT calculation. The NCI approach captures weak non-covalent interactions that are usually dwarfed by stronger interactions in many other topological analysis methods. Figure 18 depicts the non-covalent interactions in 1,1′,1,2′-tetrabromoferrocene. Regions of weak attractive interactions are illustrated in green and the brown regions illustrate strong repulsive interactions. Non-covalent interactions between the bromine atoms, both intra-ring and inter-ring, can clearly be seen. However, geometry-optimised scans of the inter-ring Br atoms dihedral angle show that the intra-ring non-covalent interactions are insufficient to stabilise the eclipsed structure and other dihedral angles are computationally more stable.

Geometry optimisations were also performed at the same DFT theory level for the isolated molecule at various dihedral angles of the inter-ring Br atoms. Key structural parameters are summarised in Table 3 for the X-ray structure and the geometry optimised structure at 0° dihedral angle.

The computational results on the isolated molecule indicate that a dihedral angle of 0° is not the lowest energy isomer. Figure 19 depicts the computed potential energy for the ring torsions relative to the Br atoms. A very shallow minimum is present at 0°, but this is too shallow to support a stable isomer and is quite high in energy compared to most other dihedral angles. Three relatively stable conformers are computed at 36°, 108° and 180°, with the torsional angle at 180° being most stable (by 1028 cm^−1^, relative to the 0° dihedral angle) where the inter-ring Br atoms are furthest away from each other. From these results it is clear that the intermolecular interactions must be responsible for the observed crystal structures.

## 3. Materials and Methods

Ferrocene (98%) was purchased from Merck (Gillingham, UK) and used as received. The haloferrocenes were legacy samples from previous work [16,17,37]. All of the compounds were recrystallised after preparation, except for the hexabromo sample, which was used as isolated from the synthesis because of its poor solubility. Elemental analysis and ^1^H NMR showed all the samples to be analytically pure. The vibrational spectra confirm that the materials are as stated. The only exception is possible residual hexane in the tetraiodoferrocene sample. However, the amount is so small it does not affect the results.

Room temperature infrared spectra (64 scans at 4 cm^−1^ resolution with eight times zero filling (to improve the peak shape)) were recorded with a Bruker (Billerica, MA, USA) Vertex 70 FTIR spectrometer spectra over the range 50–4000 cm^−1^ using the Bruker Diamond ATR accessory. Raman spectra were recorded at room temperature with the sample in a quartz cell using a Bruker (Billerica, MA, USA) FT-Raman spectrometer (64 scans at 1 cm^−1^ resolution with 500 mW laser power at 1064 nm and eight times zero filling). For the INS measurements, the sample was loaded into an In wire-sealed Al can. The sample was quenched in liquid nitrogen immediately before insertion into a closed cycle refrigerator of the indirect geometry, high-resolution, broad band spectrometer TOSCA [53,54] at the ISIS Neutron and Muon Facility [55] and was measured for 5–10 h at ~20 K. The INS spectrum of ferrocene was that previously published [24] and was downloaded from the INS database (http://wwwisis2.isis.rl.ac.uk/INSdatabase/, URL accessed 10 August 2026).

Periodic DFT calculations were carried out using the plane-wave, pseudopotential code CASTEP (versions 21.1 and 25.12) [56]. Exchange and correlation were approximated using the Perdew–Burke–Ernzerhof functional [57], with the Tkatchenko–Scheffler dispersion correction scheme [58] within the generalised gradient approximation. On-the-fly-generated norm-conserving pseudopotentials were used. The plane-wave cut-offs and the Brillouin-zone sampling of electronic states are given in Table 4 for each molecule. The equilibrium structure, an essential prerequisite for lattice dynamics calculations was obtained by Broyden–Fletcher–Goldfarb–Shanno geometry optimisation after which the residual forces were converged to zero within ±0.003 eV Å^−1^. Phonon frequencies were obtained by diagonalisation of the dynamical matrices computed using density-functional perturbation theory [59]. DFPT was also used to compute the dielectric response and the Born effective charges, and from these, the mode oscillator strength tensor and infrared absorptivity were calculated. Raman intensities were calculated by a finite displacement method [60]. The INS spectra were generated from the CASTEP output using AbINS [43].

Isolated molecule DFT calculations to investigate the conformational preference of 1,1′2,2′-tetrabromoferrocene were carried out with the Orca (version 4) computational package [61]. The DFT computations were made with the B3LYP functional, including Grimme’s atom pairwise correction with Becke–Johnson damping to account for dispersion forces [62,63,64,65]. The Ahlrich def2-TZVPP basis set [66,67,68] was used and scalar relativistic effects were accounted for by the Douglas–Kroll–Hess [69] procedure.

## 4. Conclusions

In this work we provided the first comprehensive investigation of the vibrational spectra of polyhalogenated haloferrocenes. The INS spectra above ~500 cm^−1^ are similar to that of the parent molecule, ferrocene. This can be understood because the INS spectra are dominated by hydrogen motion and the 700–1200 cm^−1^ region is where the C–H in-plane and out-of-plane modes occur. While the symmetry of the mode depends on the molecular symmetry, this is irrelevant for the INS, which only requires proton motion. This is similar in all ferrocenes that have hydrogen atoms attached to the Cp ring, so the spectra are similar. The infrared spectra of the haloferrocenes differ from that of ferrocene but are similar amongst themselves. This is a consequence of the removal of most of the symmetry elements, so all of the modes are now allowed. The Raman spectra are the most diverse: they differ from that of ferrocene and from each other. This is probably because the strongest modes are related to C-halogen modes and the form of these differ with the substitution pattern.

All the spectra differ from those of ferrocene in the region below 500 cm^−1^ because the modes that involve the halogens occur here. In addition, the modes in this region include the frustrated rotational and translational modes of the iron atom and the Cp rings. Some of these occur as whole-molecule librations and translations below 100 cm^−1^ but some become intramolecular modes of the entire molecule—ring tilts and bends—and these are mass-dependent.

## Figures and Tables

**Figure 1:**
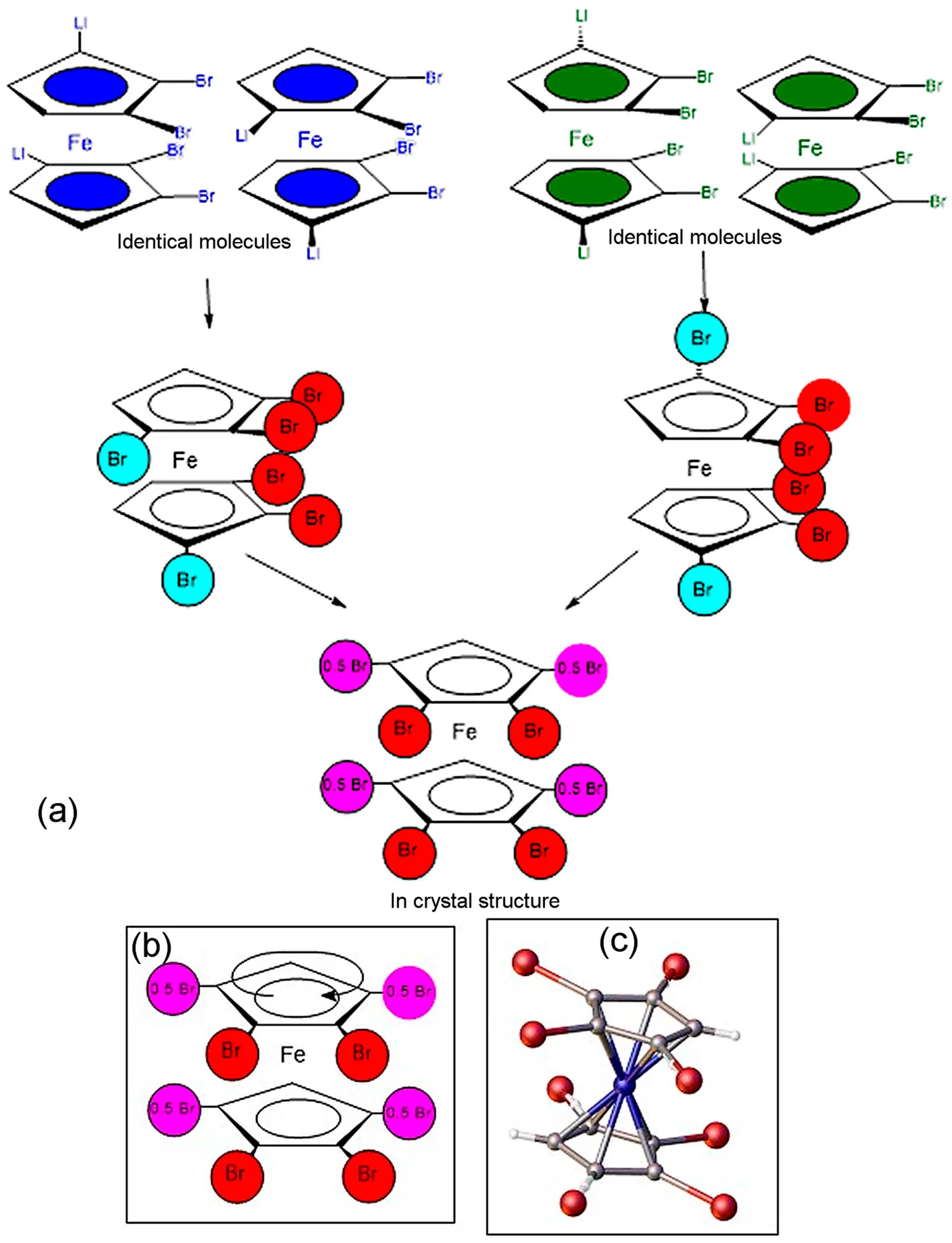
The dilithiation of 1,1′,2,2′-tetrabromoferrocene and its effect on the product hexabromoferrocene crystal structure. (**a**) All 4 possible dilithiation products are observed which are obtained from the dilithiation of 1,1′,2,2′-tetrabromoferrocene with LiTMP/TMEDA in non-polar solvents (only 2 of these are unique, as shown by the blue and green colours). Following quenching (where lithium was replaced by bromine), partial occupancy results in four of the bromine sites in the crystal structures; 4 bromine atoms (red) have unit occupancy, and 4 bromine atoms (light blue) have ca 50% occupancy (**b**). In this crystal structure (**c**) the cyclopentadienyl rings are staggered. In the work discussed later, we examine the eclipsed structure of this compound which was prepared independently following the initial work.

**Figure 2:**
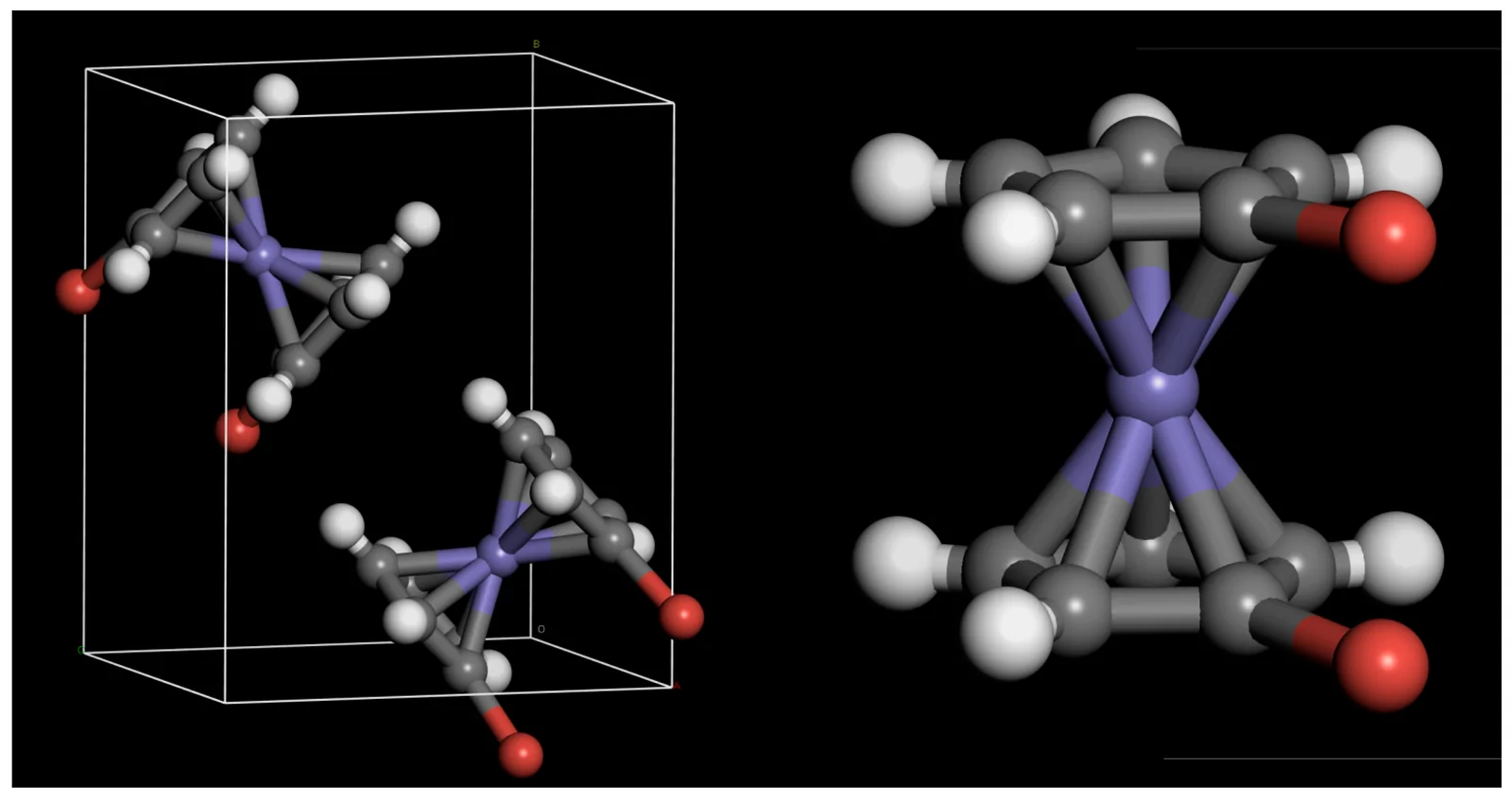
Structure of 1,1′-dibromoferrocene. **Left**: complete primitive unit cell. **Right**: one molecule showing the eclipsed geometry of the Br atoms and approximate *C*_2v_ symmetry. Key: C = grey; H = white; Br = rust red; Fe = purple.

**Figure 3:**
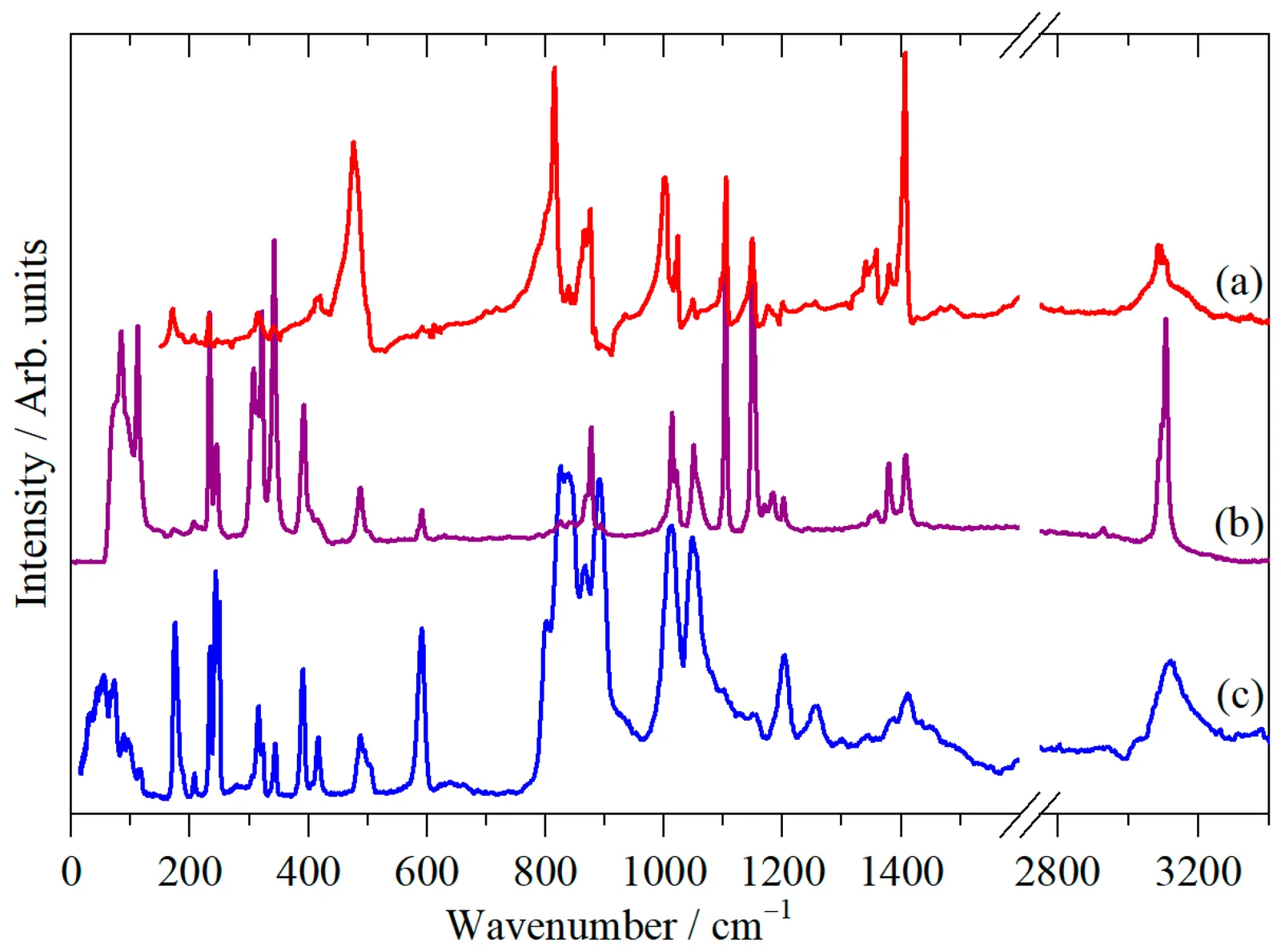
Vibrational spectra of 1,1′-dibromoferrocene: (**a**) infrared, (**b**) Raman and (**c**) INS.

**Figure 4:**
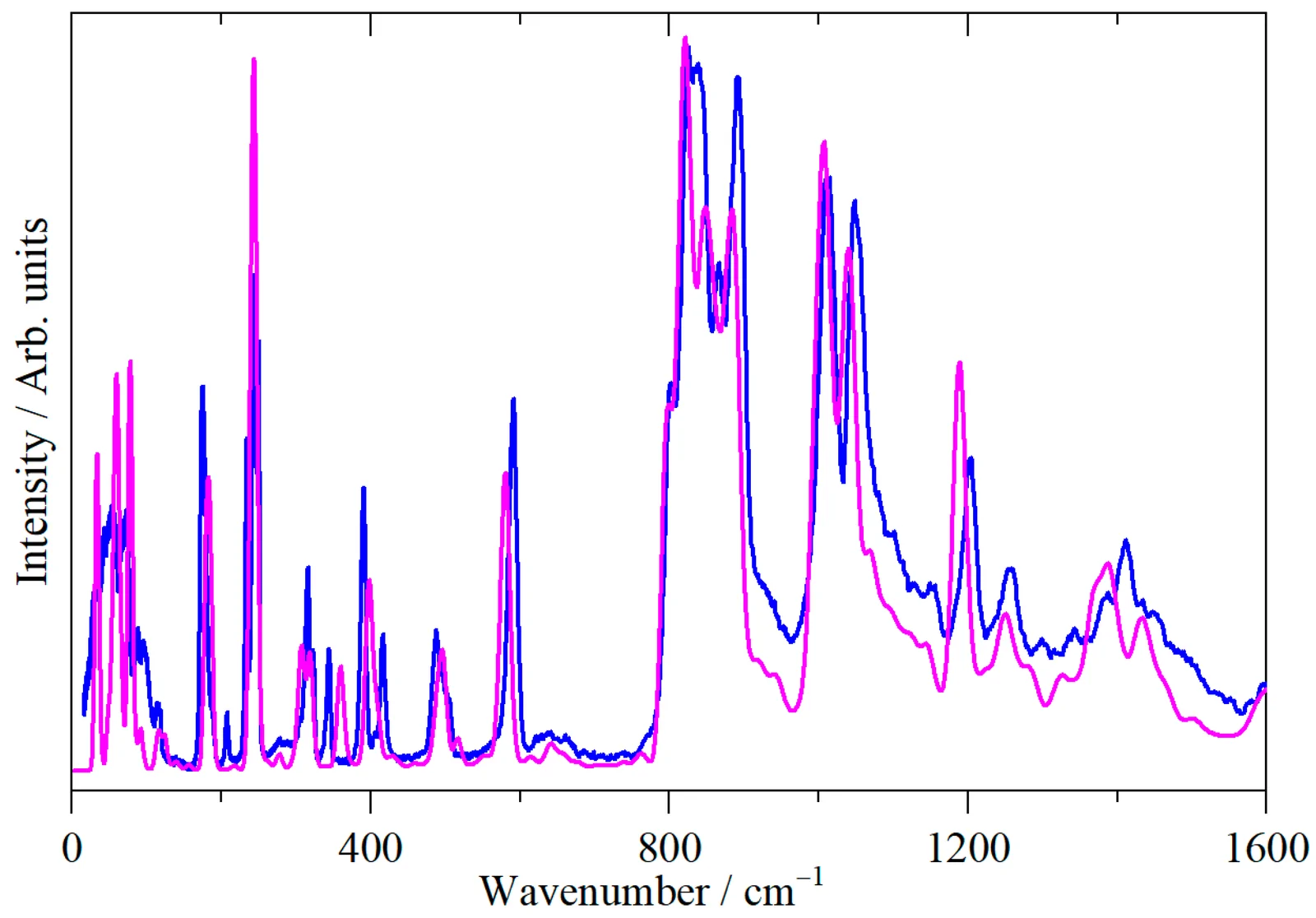
Comparison of observed (blue) and calculated (magenta) INS spectra of 1,1′-dibromoferrocene.

**Figure 5:**
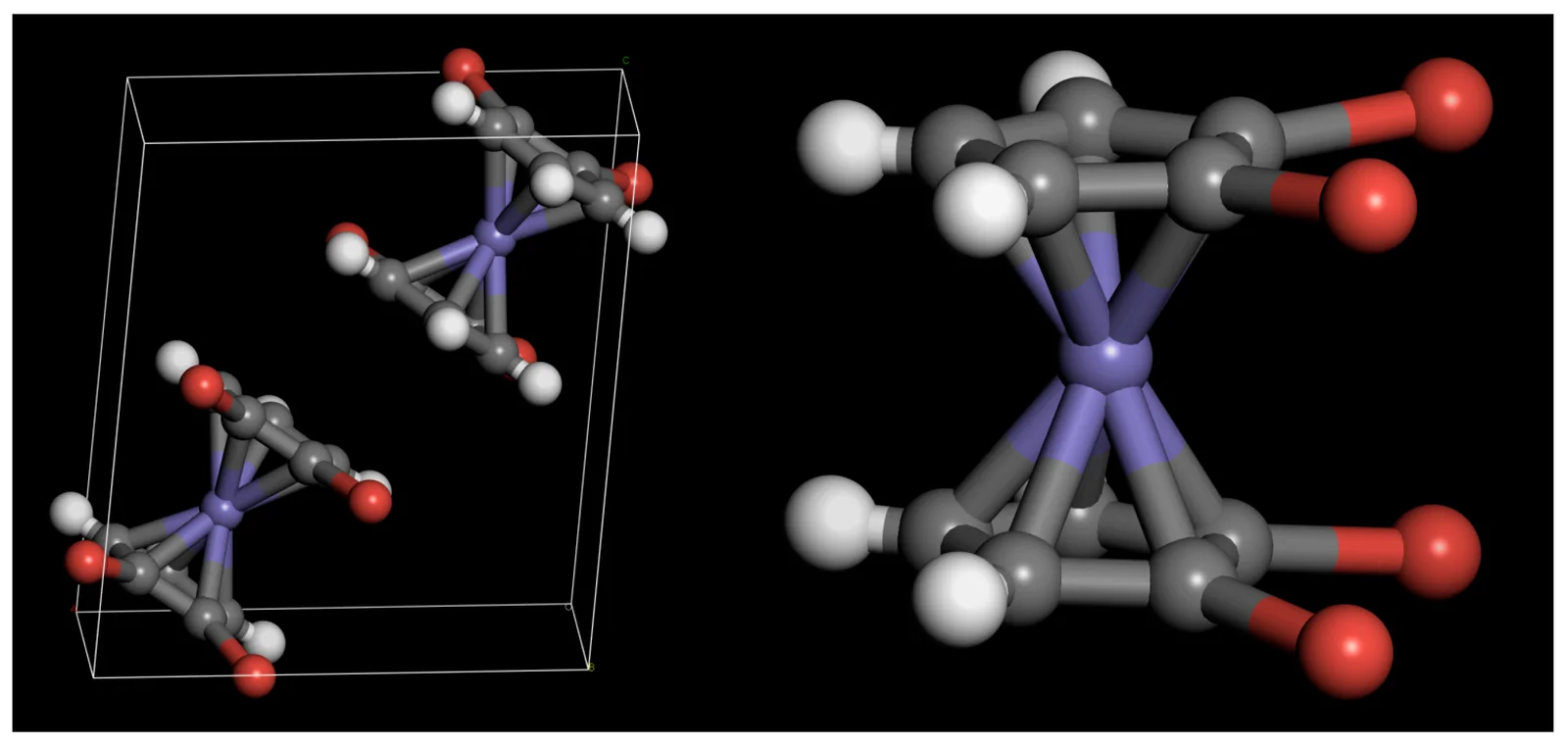
Structure of 1,1′,2,2′-tetrabromoferrocene. **Left**: complete primitive unit cell. **Right**: one molecule showing the eclipsed geometry of the Br atoms and approximate *C*_2v_ symmetry. Key: C = grey; H = white; Br = rust red; Fe = purple.

**Figure 6:**
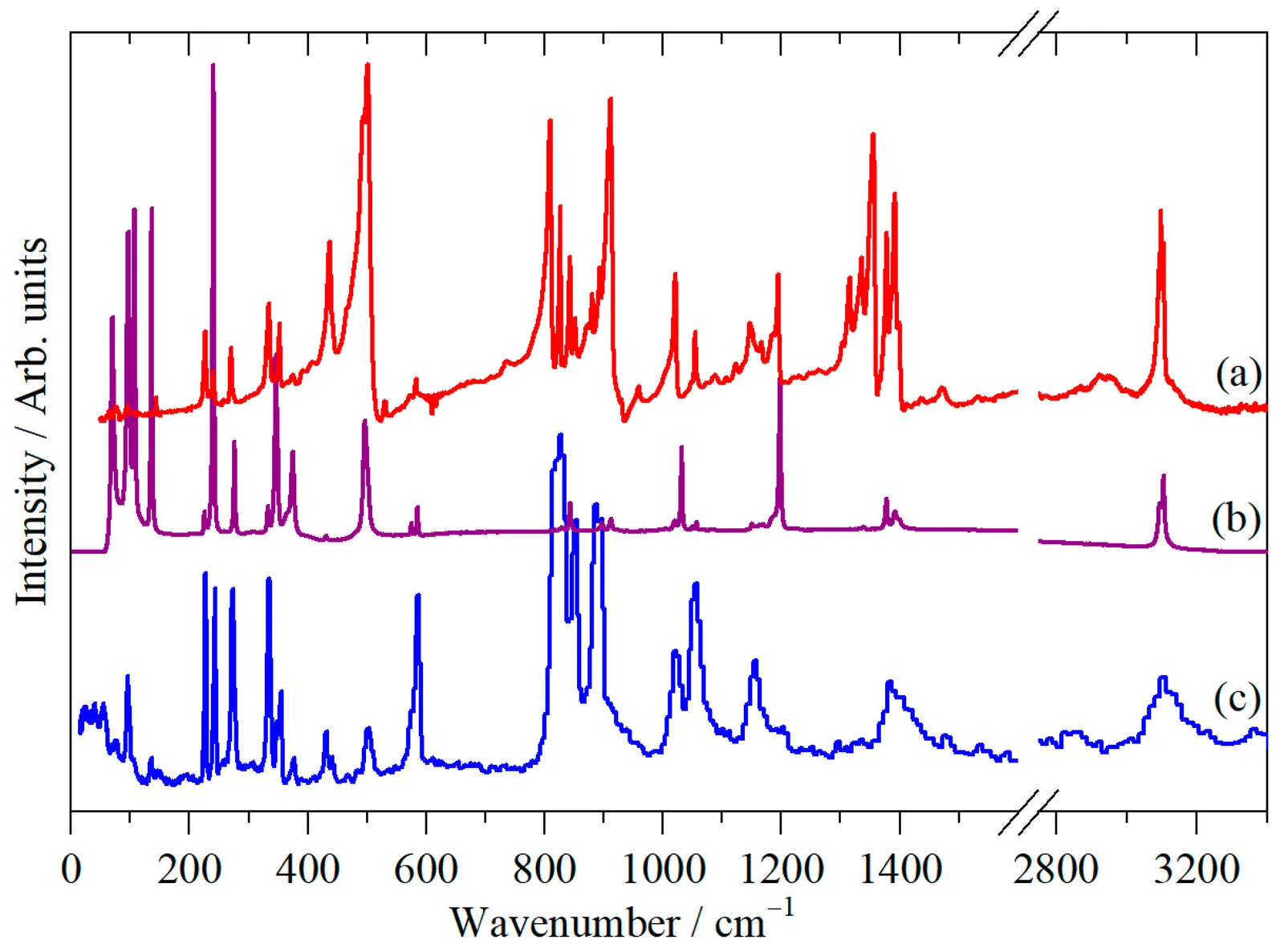
Vibrational spectra of 1,1′,2,2′-tetrabromoferrocene: (**a**) infrared, (**b**) Raman and (**c**) INS.

**Figure 7:**
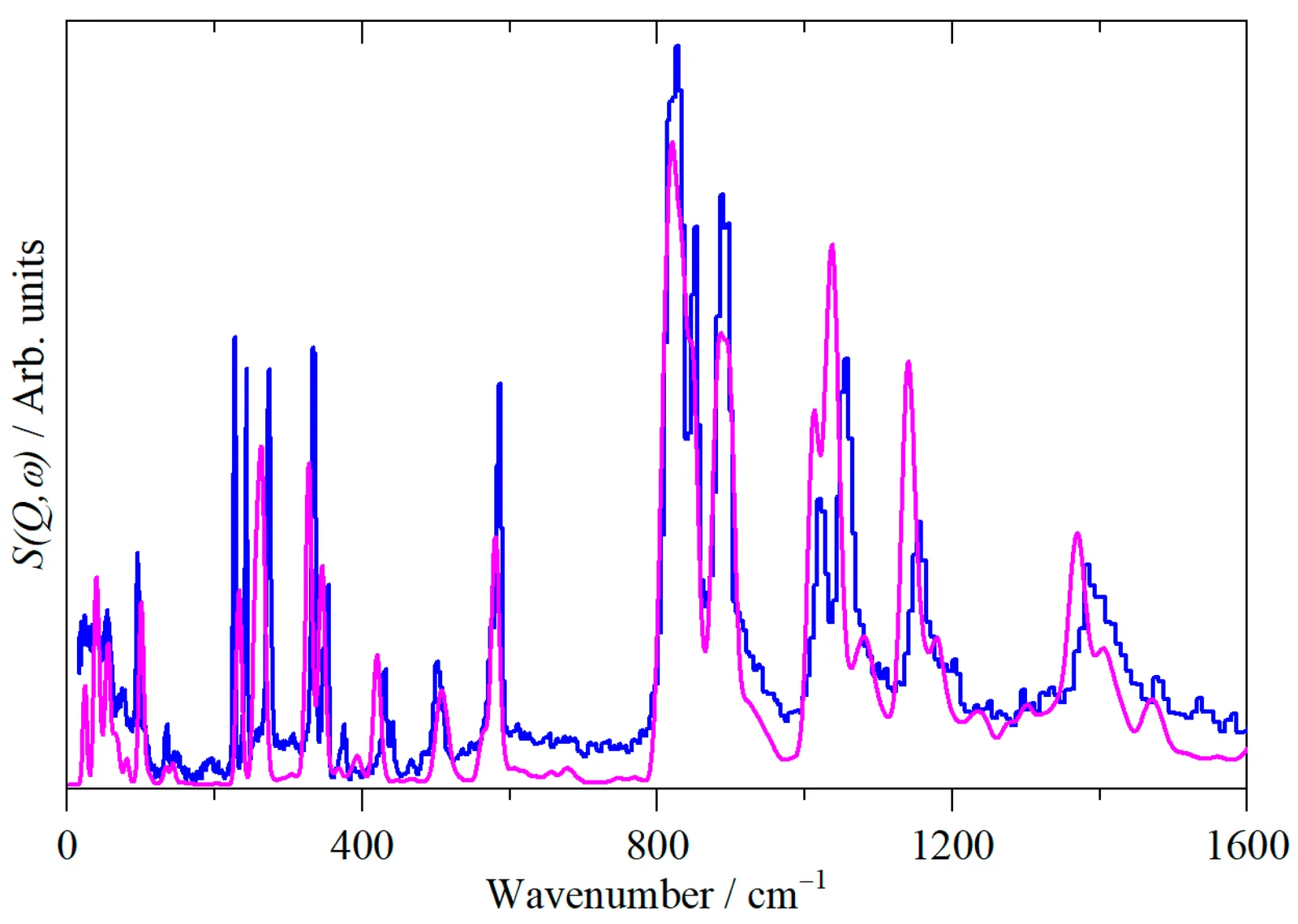
Comparison of observed (blue) and calculated (magenta) INS spectra of 1,1′,2,2′-tetrabromoferrocene.

**Figure 8:**
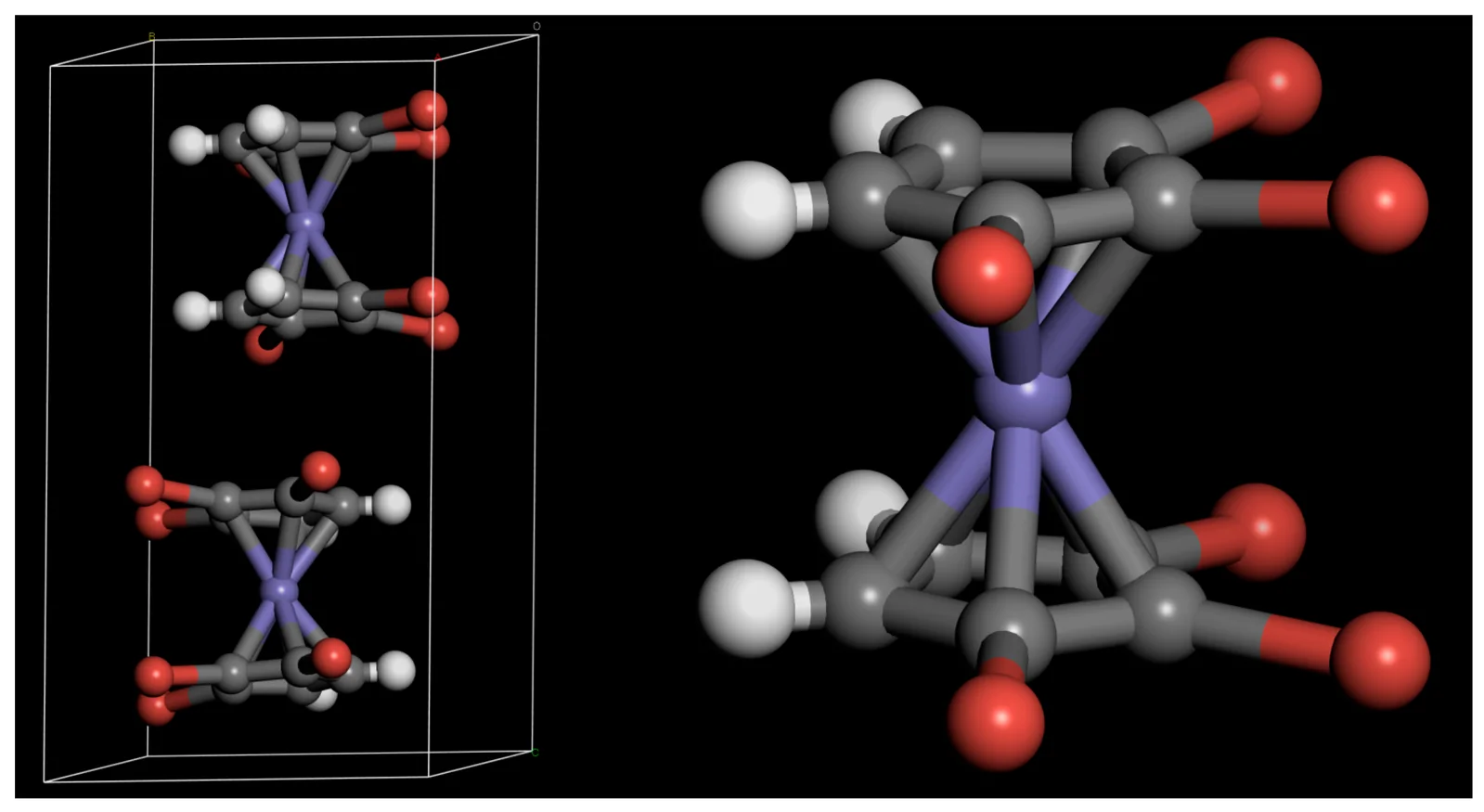
Structure of 1,1′,2,2′,3,3′-hexabromoferrocene. **Left**: complete primitive unit cell. **Right**: one molecule showing the eclipsed geometry of the Br atoms and approximate *C*_2v_ symmetry. Key: C = grey; H = white; Br = rust red; Fe = purple.

**Figure 9:**
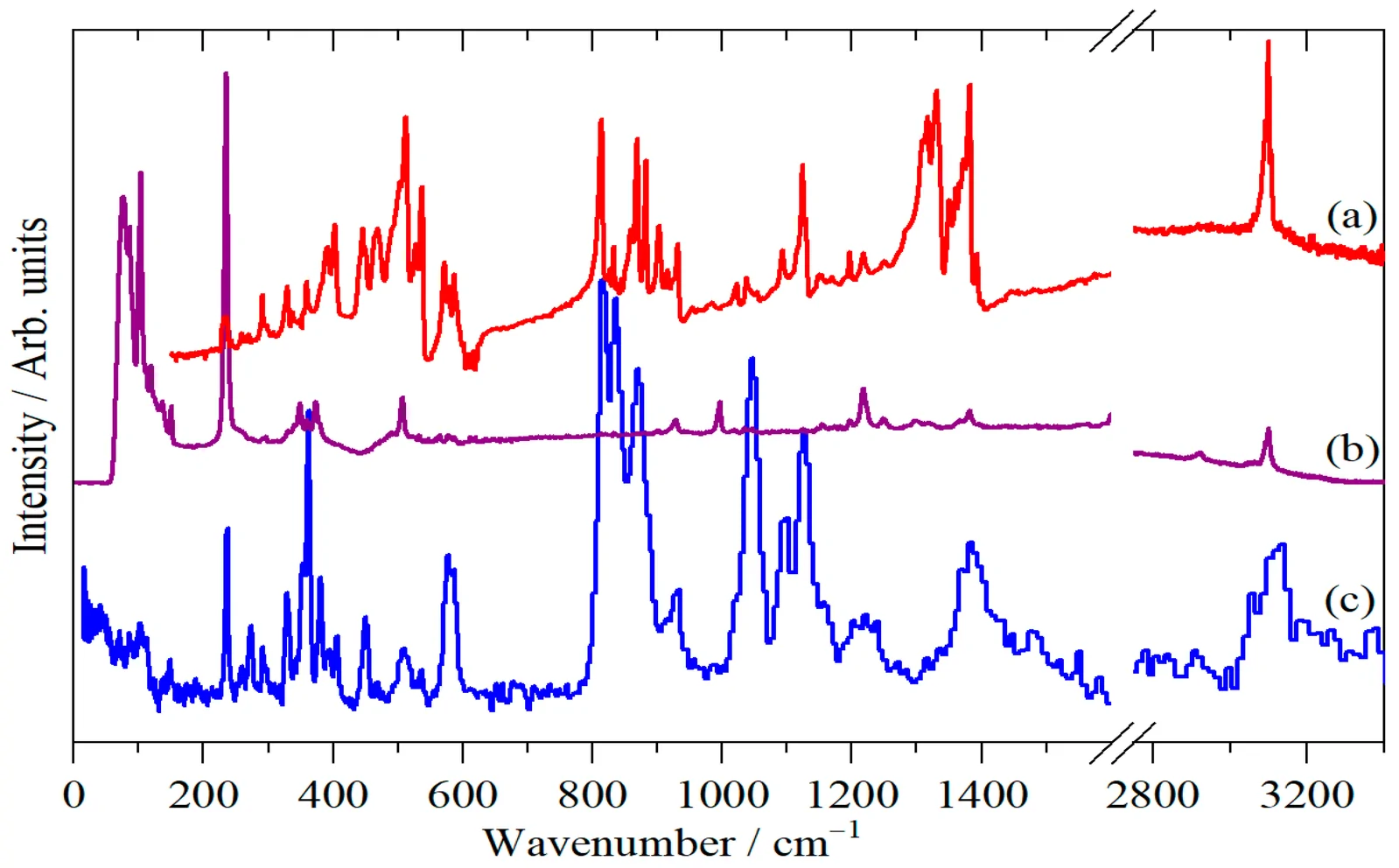
Vibrational spectra of 1,1′,2,2′,3,3′-hexabromoferrocene: (**a**) infrared, (**b**) Raman and (**c**) INS.

**Figure 10:**
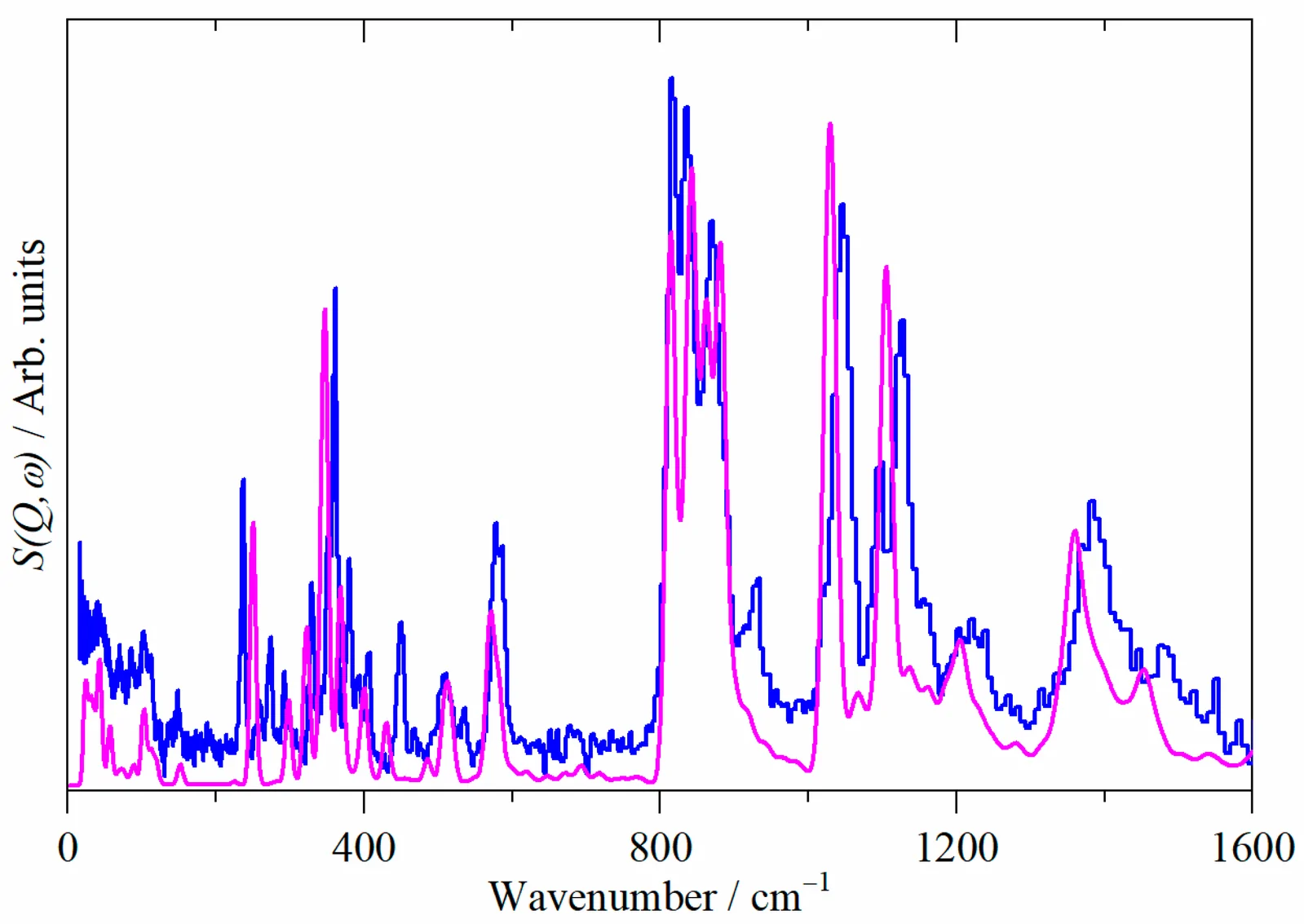
Comparison of observed (blue) and calculated (magenta) INS spectra of 1,1′,2,2′,3,3′-hexabromoferrocene.

**Figure 11:**
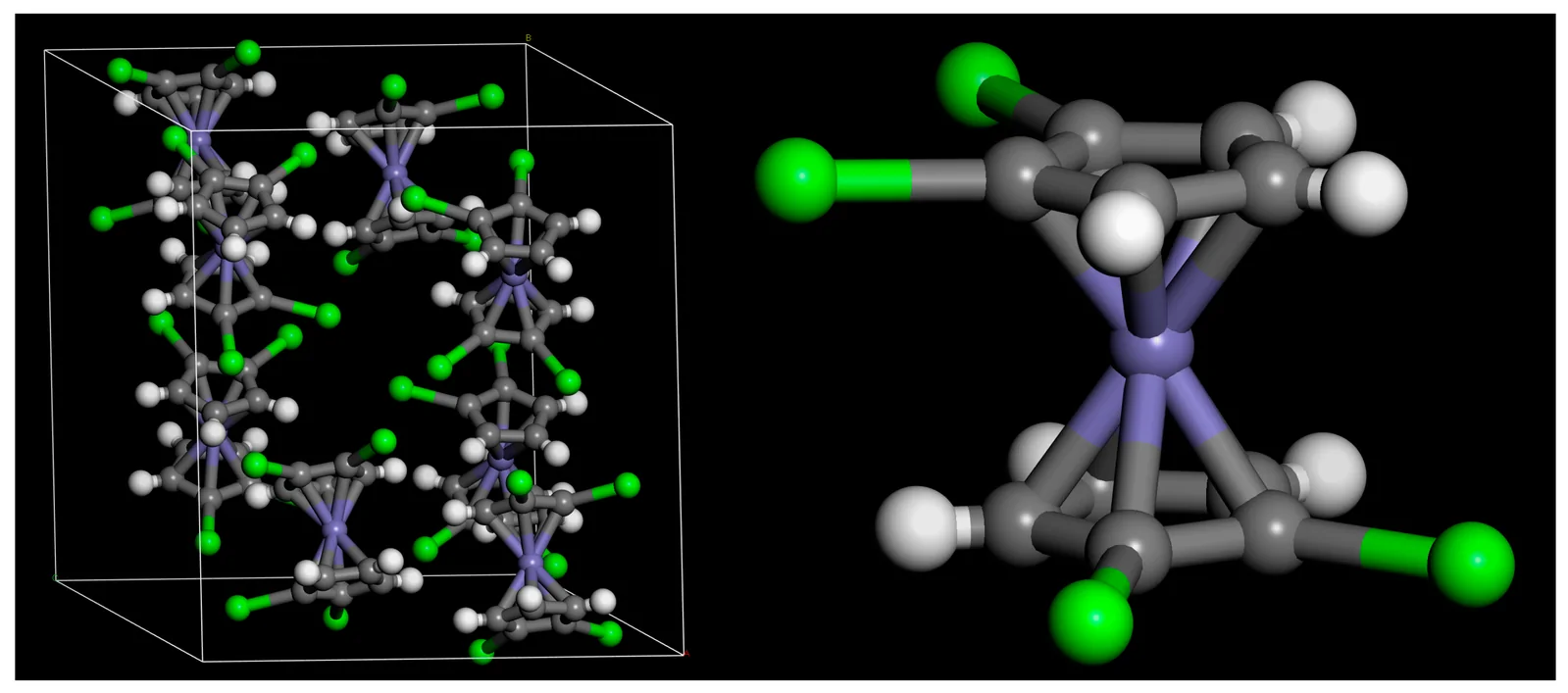
Structure of 1,1′,2,2′-tetraiodoferrocene. **Left**: complete primitive unit cell. **Right**: one molecule showing the staggeredgeometry of the I atoms and approximate *C*_2_ symmetry. Key: C = grey; H = white; I = green; Fe = purple.

**Figure 12:**
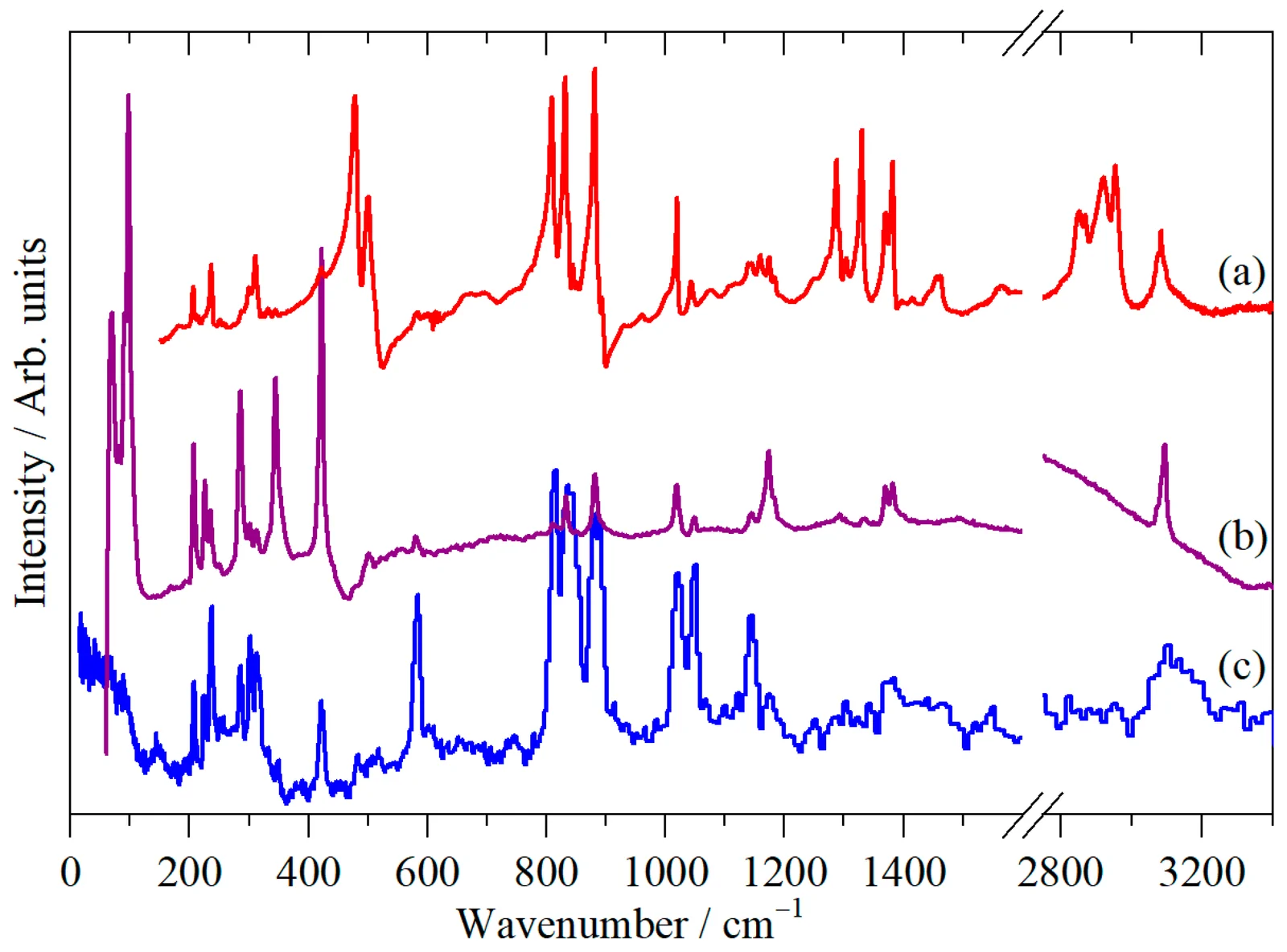
Vibrational spectra of 1,1′,2,2′-tetraiodoferrocene: (**a**) infrared, (**b**) Raman and (**c**) INS.

**Figure 13:**
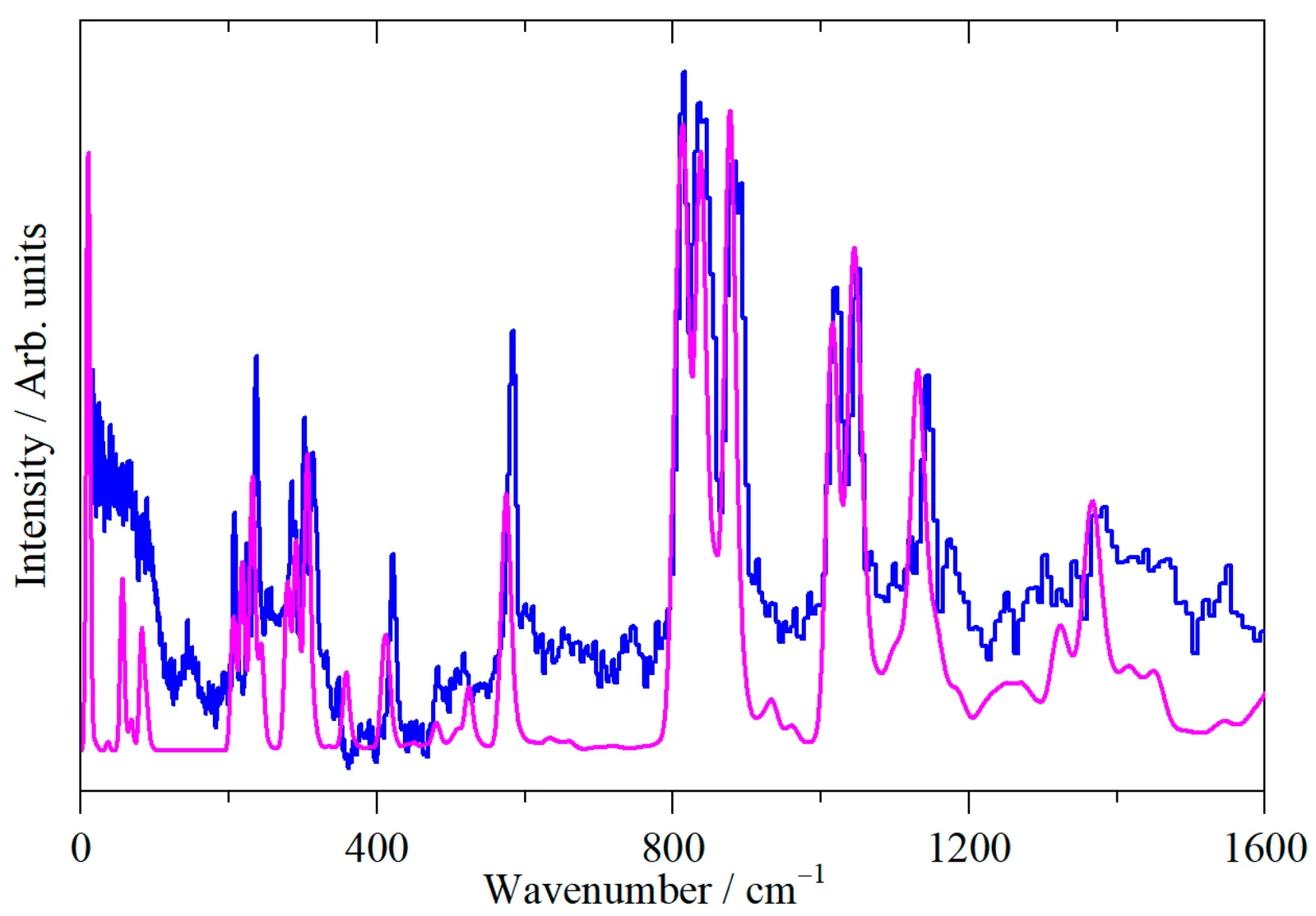
Comparison of observed (blue) and calculated (magenta) INS spectra of 1,1′,2,2′-tetraiodoferrocene.

**Figure 14:**
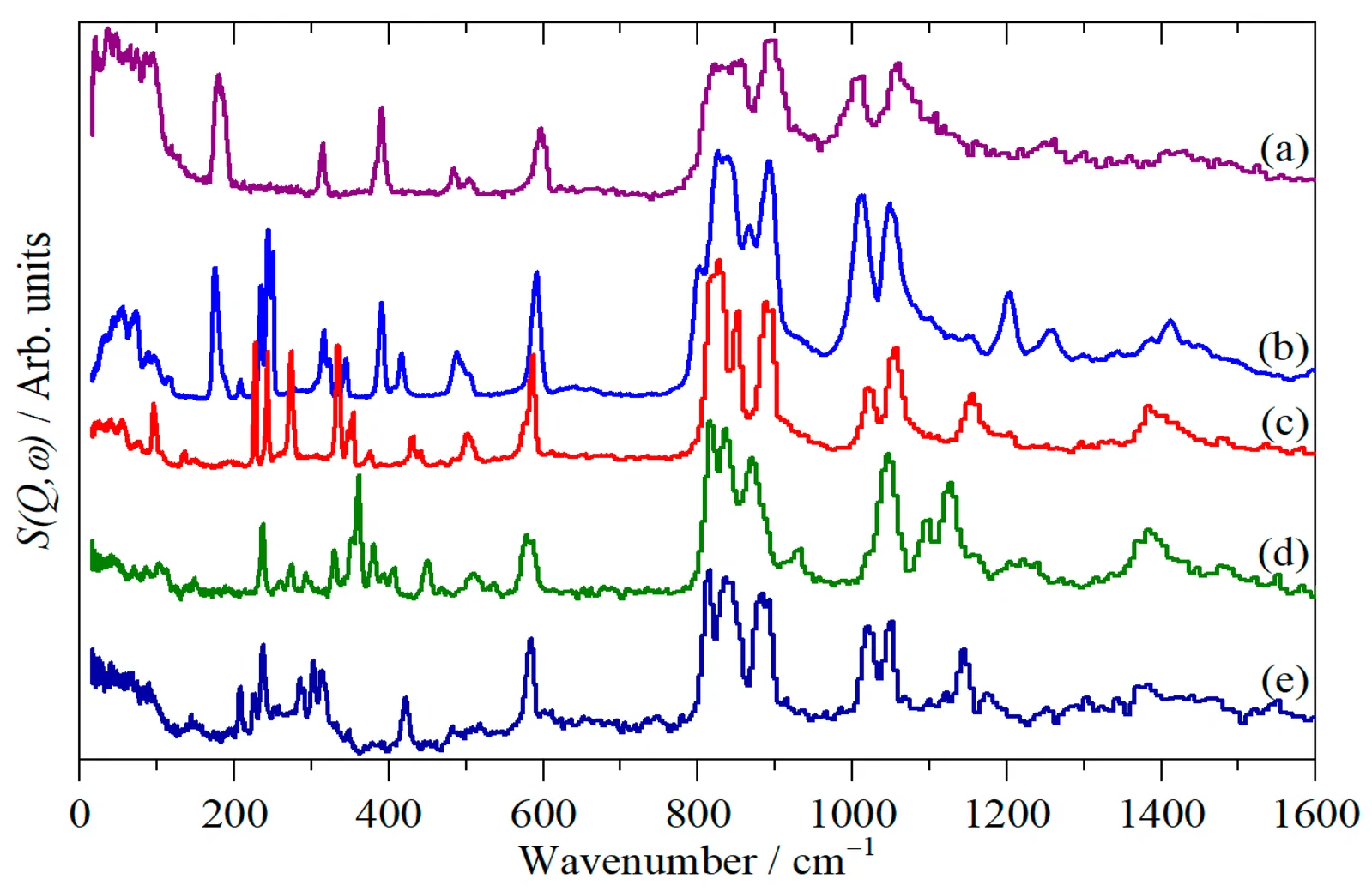
Comparison of INS spectra of: (**a**) ferrocene, (**b**) 1,1′-dibromoferrocene, (**c**) 1,1′,2,2′-tetrabromoferrocene, (**d**) 1,1′,2,2′,3,3′-hexabromoferrocene and (**e**) 1,1′,2,2′-tetraiodoferrocene.

**Figure 15:**
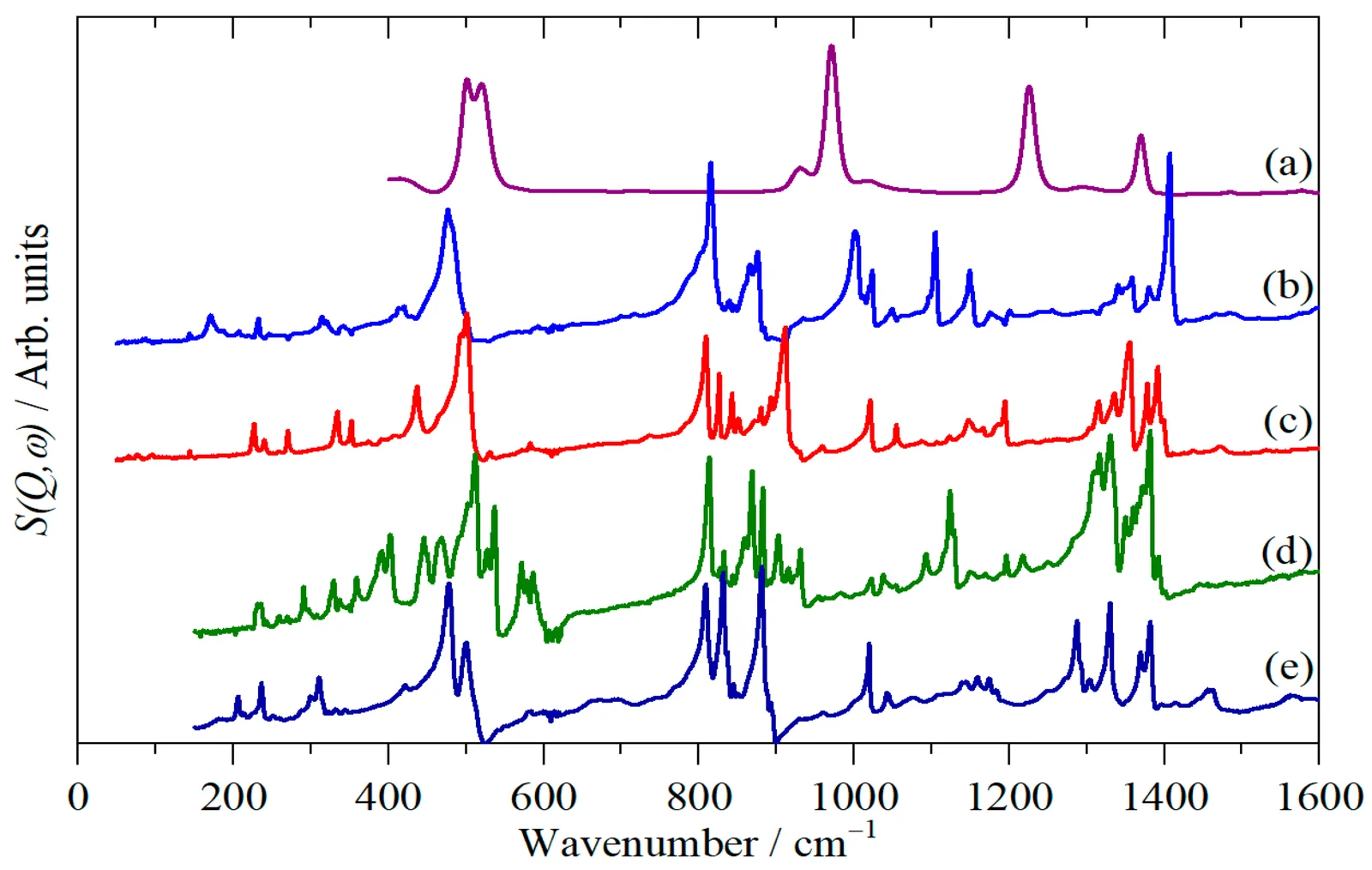
Comparison of infrared spectra of: (**a**) ferrocene, (**b**) 1,1′-dibromoferrocene, (**c**) 1,1′,2,2′-tetrabromoferrocene, (**d**) 1,1′,2,2′,3,3′-hexabromoferrocene and (**e**) 1,1′,2,2′-tetraiodoferrocene.

**Figure 16:**
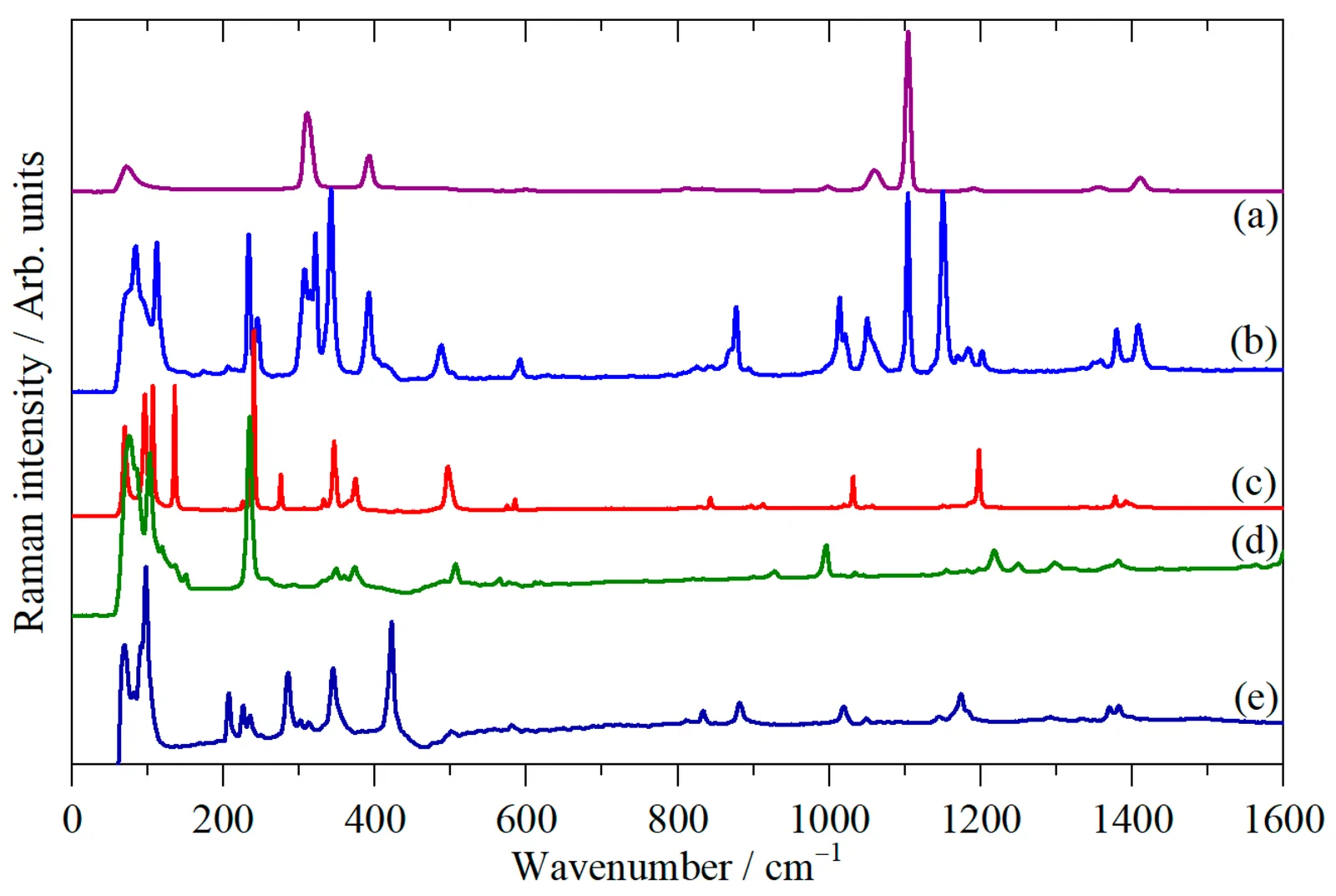
Comparison of Raman spectra of: (**a**) ferrocene, (**b**) 1,1′-dibromoferrocene, (**c**) 1,1′,2,2′-tetrabromoferrocene, (**d**) 1,1′,2,2′,3,3′-hexabromoferrocene and (**e**) 1,1′,2,2′-tetraiodoferrocene.

**Figure 17:**
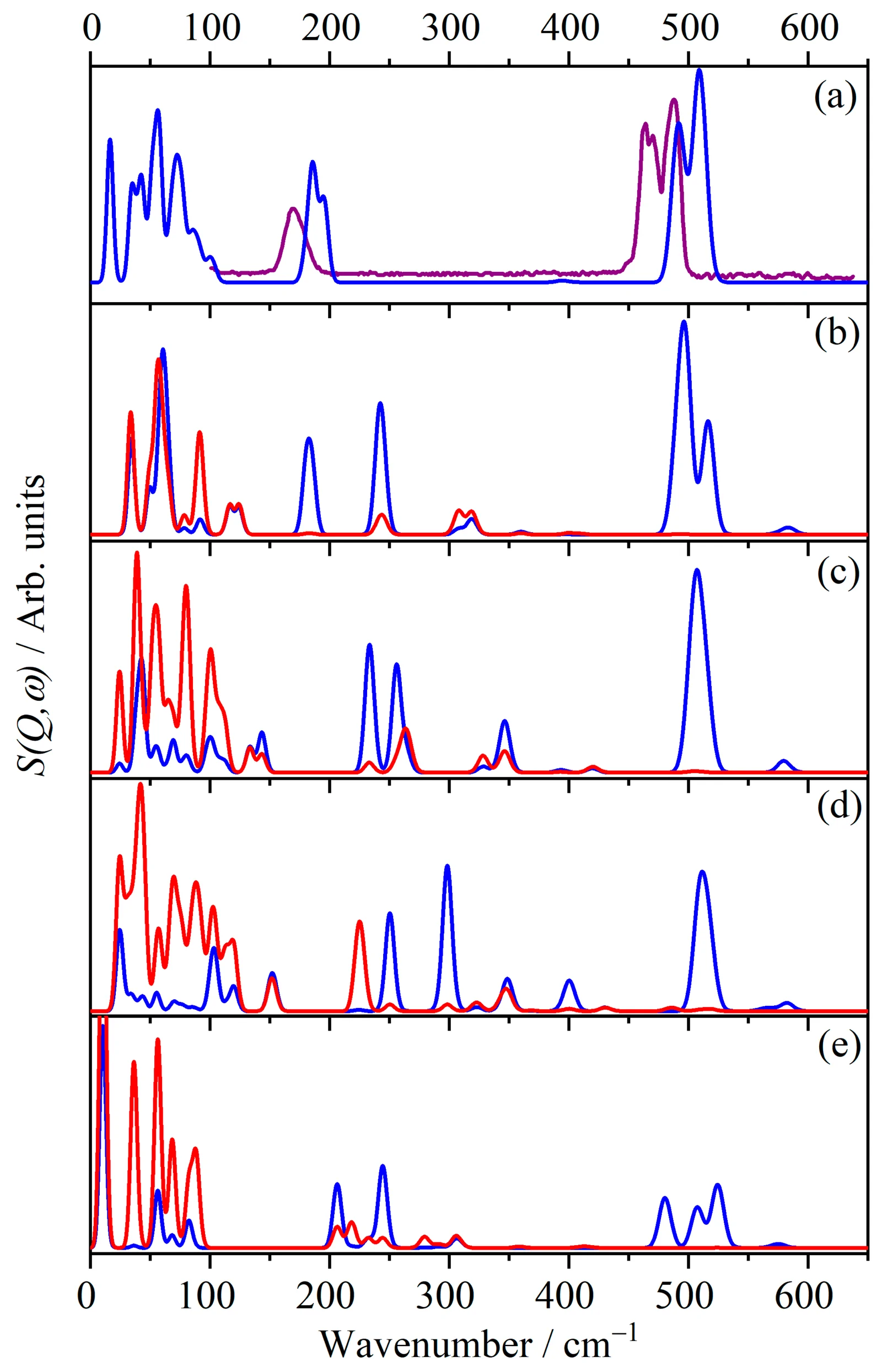
(**a**) NRVS spectrum of ferrocene (purple) [25] and calculated Fe-only INS spectrum of ferrocene (blue). Panels (**b**–**e**) show the calculated Fe-only (blue) and halogen-only (red) INS spectra of: (**b**) 1,1′-dibromoferrocene, (**c**) 1,1′,2,2′-tetrabromoferrocene, (**d**) 1,1′,2,2′,3,3′-hexabromoferrocene and (**e**) 1,1′,2,2′-tetraiodoferrocene. The NRVS spectrum in part (**a**) is reproduced from [25] with permission of WILEY-VCH-Verlag GmbH.

**Figure 18:**
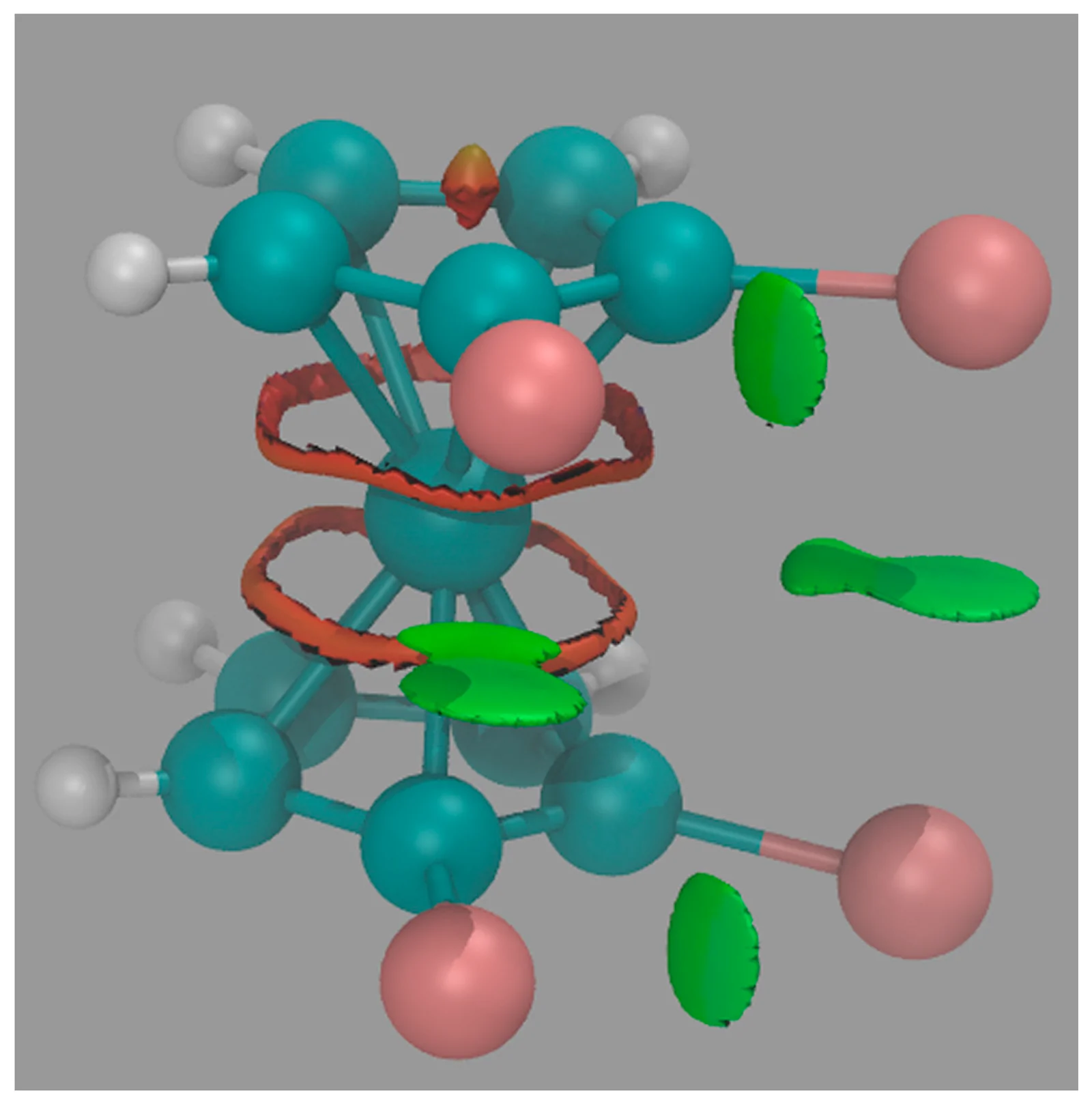
Non-covalent interactions in 1,1′,1,2′-tetrabromoferrocene, as visualised using NCIPLOT [52]. Weak attractive regions are depicted in green and strong repulsive regions are depicted in brown.

**Figure 19:**
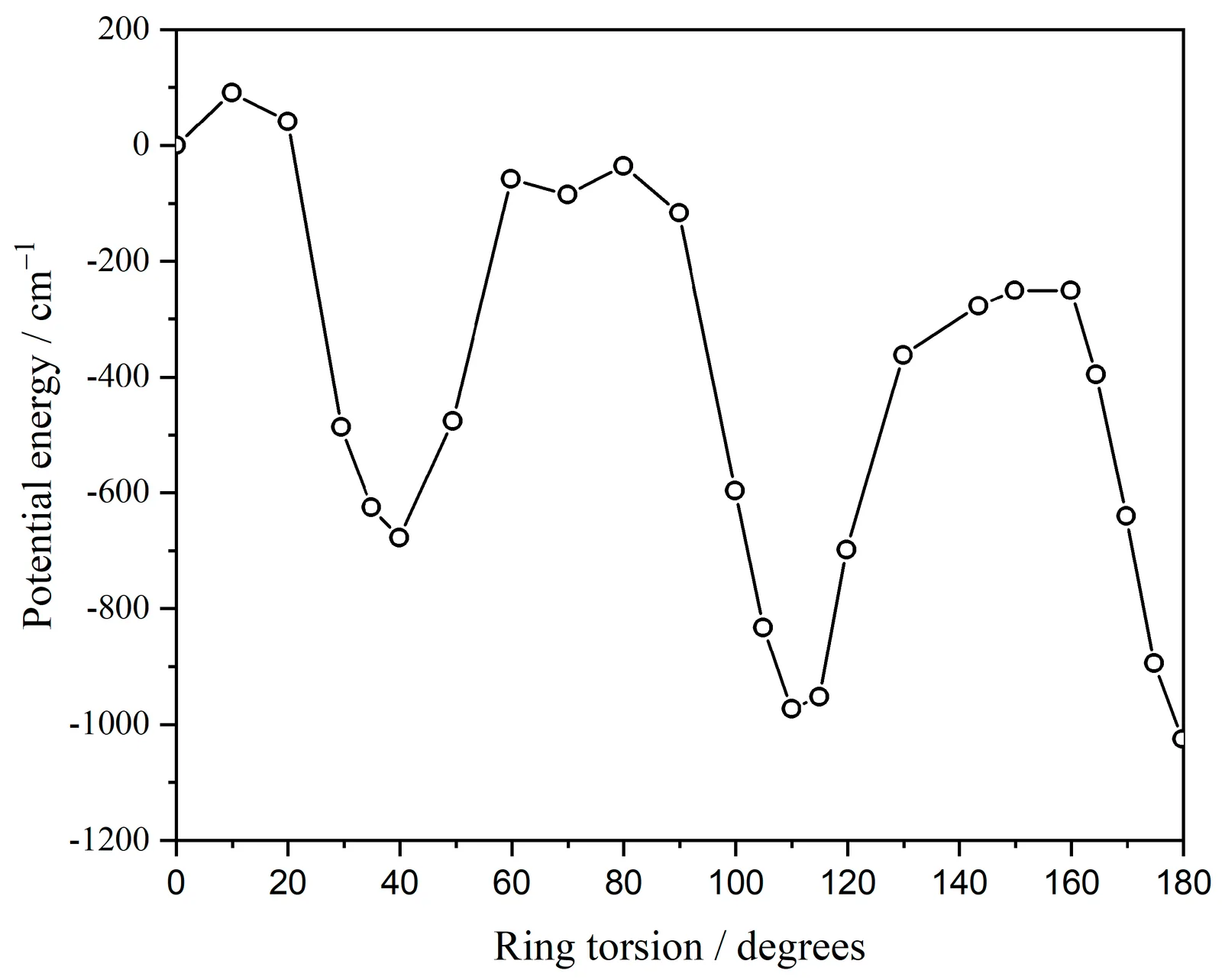
Ring torsional potential energy for 1,1′2,2′-tetrabromoferrocene computed from DFT (Orca with the B3LYP functional and the Ahlrich def2-TZVPP basis set). The ring torsions are defined in terms of the dihedral angles of the inter-ring Br atoms. The lines are a guide to the eye.

**Table 1:** Fe-modes of ferrocene and haloferrocenes.

Molecule	Ring Bend ν_22_ /cm^−1^	Symmetric Stretch ν_4_ /cm^−1^	Fe–Ring Tilt ν_16_ /cm^−1^	Asymmetric Stretch ν_11_ /cm^−1^	Fe–Ring Tilt ν_21_ /cm^−1^
Ferrocene	179	315	391	484	505
	(181–196)	(330–337)	(390–405)	(489–495)	(505–513)
1,1′-Dibromoferrocene	175 ^1^	344	391,417	488	496,506
	(180,186,240–246)	(359–360)	(396,398,401–409)	(490,498)	(498–516)
1,1′,2,2′-Tetrabromoferrocene	228,243	376	432,442	502	502
	(233,235,255,256)	(391,395)	(418–423)	(509,516)	(501–507)
1,1′,2,2′,3,3′-Hexabromoferrocene	237,273,292	392	408,450	494	509
	(250,251,299,299)	(395,399)	(400,402,430,430)	(511,515)	(485–520)
1,1′,2,2′-Tetraiodoferrocene	208,255	348	422	518	481,508
	(206,244) ^2^	(358)	(410,415)	(524)	(480,507)

^1^ The observed transition energies are given for the experimental values and the rangeof the factor group components for the calculated values in parentheses below the experimental value. ^2^ Only a single molecule was calculated for this molecule, so there are no factor group components.

**Table 3:** Experimental [16] and computed geometries of 1,1′2,2′-tetrabromoferrocene. Computed geometries were constrained to ‘cis’ zero degrees ring torsion of the Br atoms. Lengths (*r*) are in Å. The calculations were carried out using Orca with the B3LYP functional and the Ahlrich def2-TZVPP basis set.

	Experiment	Computed
*r*(Fe-Cp)	1.652	1.643
*r*(C-Br)	1.888 and 1.878	1.846 and 1.847
*r*(Br-Br) (inter-ring)	3.713	3.647
*r*(Br-Br) (intra-ring)	3.610	3.631

**Table 4:** DFT parameters for ferrocene and the halogenated ferrocenes. For all of the calculations, the CASTEP programme with the PBE functional, TS dispersion correction and on-the-fly-generated pseudopotentials were used.

Molecule	Cut-Off Energy /eV	Monkhorst-Pack Grid	No. of *k*-Points	Convergence /eV Å^−1^
Ferrocene	1440	8 × 5 × 6	120	0.0040
1,1′-Dibromoferrocene	1440	8 × 5 × 6	120	0.0041
1,1′,2,2′-Tetrabromoferrocene	1440	8 × 5 × 6	72	0.0029
1,1′,2,2′,3,3′-Hexabromoferrocene	1440	8 × 6 × 4	96	0.0050
1,1′,2,2′-Tetraiodoferrocene	1440	8 × 5 × 6	72	0.0022

## Data Availability

The original data presented in this study are openly available in eData, the STFC Research Data Repository at https://doi.org/10.5286/edata/985 (accessed on 10 August 2026).
